# Integrin-Linked Kinase (ILK) Plays an Important Role in the Laminin-Dependent Development of Dorsal Root Ganglia during Chicken Embryogenesis

**DOI:** 10.3390/cells10071666

**Published:** 2021-07-02

**Authors:** Ewa Mrówczyńska, Antonina Joanna Mazur

**Affiliations:** Department of Cell Pathology, Faculty of Biotechnology, University of Wroclaw, 50-383 Wroclaw, Poland

**Keywords:** integrin-linked kinase (ILK), laminin, peripheral nervous system (PNS), dorsal root ganglion (DRG), axonal outgrowth, Schwann cell precursors (SCPs), chicken, embryogenesis, extracellular matrix (ECM)

## Abstract

Integrin-linked kinase (ILK) is mainly localized in focal adhesions where it interacts and modulates the downstream signaling of integrins affecting cell migration, adhesion, and survival. The interaction of dorsal root ganglia (DRG) cells, being part of the peripheral nervous system (PNS), with the extracellular matrix (ECM) via integrins is crucial for proper PNS development. A few studies have focused on ILK’s role in PNS development, but none of these have focused on chicken. Therefore, we decided to investigate ILK’s role in the development of *Gallus gallus domesticus’s* DRG. First, using RT-PCR, Western blotting, and in situ hybridization, we show that *ILK* is expressed in DRG. Next, by immunocytochemistry, we show ILK’s localization both intracellularly and on the cell membrane of DRG neurons and Schwann cell precursors (SCPs). Finally, we describe ILK’s involvement in multiple aspects of DRG development by performing functional experiments in vitro. IgG-mediated interruption of ILK’s action improved DRG neurite outgrowth, modulated their directionality, stimulated SCPs migration, and impacted growth cone morphology in the presence of laminin-1 or laminin-1 mimicking peptide IKVAV. Taken together, our results show that ILK is important for chicken PNS development, probably via its exposure to the ECM.

## 1. Introduction

Dorsal root ganglia (DRG) are indispensable components of the peripheral nervous system (PNS), transferring neural messages from skin, muscles, and internal organs to the spinal cord and brain [1,2]. DRG are composed of a body cell mass (BCM), which encompasses somas surrounded by satellite cells, as well as extending neurites with supporting Schwann cell precursors (SCPs) [2]. During vertebrate’s development, DRG develop and extend long-distance axons accompanied by SCPs, which are both involved in the further transfer of signals. Later, SCPs differentiate to Schwann cells and, in the case of trunk DRG, also to melanocytes [3].

Extracellular matrix (ECM) proteins such as laminins are crucial during PNS development [4]. Laminins are large glycoproteins consisting of three chains α, β, and γ, which include various binding sites for the cell receptors [5,6]. They are responsible for the modulation of laminins’ biological activities, such as cell adhesion, growth, differentiation, and migration [7,8,9]. Laminins stimulate DRG development [10,11], while laminin-1 was found to most efficiently stimulate sensory neurons’ extension [12]. The active binding site of laminin responsible for the stimulation of neurite outgrowth, but also cell attachment and migration, is the pentapeptide IKVAV (Ile-Lys-Val-ala-Val) localized on the laminin α chain [9,13]. Integrins, being the receptors for ECM proteins, are responsible for the transduction of ECM-cell signals, thereby triggering signal transduction and modulating motility, adhesion, proliferation, differentiation, and survival, which is crucial for proper PNS development [14]. IKVAV, as a laminin-derived peptide, binds directly to the α3β1, α4β1, and α6β1 integrins [15,16].

Integrin-linked kinase (ILK) is a 52-kDa protein with putative serine-threonine kinase activity that interacts with the cytoplasmic β1 and β3 tails of integrins at focal adhesion sites and hence mediates ECM-actin cytoskeleton signaling [17]. ECM-cell contacts, neurotrophic factors and insulin stimulate ILK activity [18,19]. Therefore, since ILK was discovered [17], it has become a focus of research wherever cellular processes like adhesion, migration, or proliferation are studied. ILK’s importance during embryonic development has been described, noting the lethality in mice embryos with ILK-conditional knockout [20]. ILK takes part in neuronal polarization [21], radial sorting of Schwann cells [22], oligodendrocyte differentiation, and myelination of cells of the central nervous system (CNS) [23]. Moreover, ILK is essential for neurite outgrowth [19], proliferation, and adhesion of keratinocytes and chondrocytes [24,25,26]. Beyond ILK’s crucial role in development, its strong expression has also been correlated with advanced tumor stage and poor prognosis in various cancers [27,28,29]. Additionally, ILK-depleted melanoma cells exhibited defected adhesion, spreading, migration, and invasion [30].

Considering that laminin-integrin interactions are critical during PNS development and that ILK is associated with integrins, our study aims to deepen the knowledge about ILK’s role in PNS formation at the stage of DRG, as there are still gaps in understanding its function in this area, especially that no work addressing this topic has so far been done in chicken embryos. First, we determined *ILK’s* expression at mRNA and protein levels in DRG of HH34 chicken embryos grown without and upon stimulation of DRG neurite outgrowth with laminin-1. The next objective of this study was to investigate the detailed localization of ILK in DRG. For the first time, we show ILK’s presence inside DRG neuronal cell bodies, neurites, growth cones, and the supporting SCPs, and on these cells’ surfaces. However, we show that ILK is not secreted to the medium. Since ILK is exposed to the ECM, we decided to alter ILK’s function using specific anti-ILK antibodies to prove the importance of ILK for the development and functioning of DRG. This generated intriguing data proving that ILK plays a role in laminin-1-mediated DRG neurite outgrowth. Moreover, ILK influences axonal extension and directionality as well as SCPs migration. Additionally, disruption of ILK’s function resulted in changes in growth cone morphology. Interestingly, all these changes depended on the substratum type (laminin-1 or its peptide IKVAV) and its administration method.

## 2. Materials and Methods

### 2.1. Animals and Ethics Statement

Following the Polish and European acts on the Protection of Animals Used for Scientific or Educational Purposes, no formal approvals were required to perform these experiments involving chicken *Gallus gallus domesticus* embryos ((stage HH21 (E3.5), HH34 (E8), HH36 (E10) according to the Hamburger and Hamilton classification [31]). All procedures were accomplished using as few chicken embryos as possible and minimizing any potential pain, distress, and suffering. Fertilized chicken eggs were incubated in an egg incubator at 37 °C and 80% humidity until the desired stages of chicken embryo development were reached.

### 2.2. RNA Isolation and Reverse-Transcription Polymerase Chain Reaction (RT-PCR)

Each RNA sample was prepared from the culture of 30 DRG. DRG were homogenized with vortexing, and then RNA was extracted and purified according to the phenol/chloroform extraction method [32]. The High-Capacity cDNA Reverse Transcription Kit (MP Biomedicals, Eschwege, Germany) was used to convert 0.5 μg of RNA to cDNA per sample, as the manufacturer recommended. Subsequently, PCR was performed using 1 μL of cDNA mixed with 2 × PCR Master Mix (Thermo Fisher Scientific, Warsaw, Poland) and primers designed for *ILK* isoforms and *ACTB* (listed in Supplementary Table S1). PCRs were performed under following conditions: initial denaturation 95 °C, 4 min; 30 cycles of: denaturation (95 °C, 30 s), annealing (Ta, 30 s), and extension (72 °C, 45 s); final extension (72 °C, 10 min); cooling (8 °C, ∞). The PCR products were next run on 2% agarose gels in Tris-acetate-EDTA (TAE) buffer and visualized with ChemiDocTM MP System and ImageLab 4.0 software from Bio-Rad (Hercules, CA, USA). GeneRuler 100 bp Plus DNA Ladder (Thermo Fisher Scientific, Warsaw, Poland) was used as a marker.

### 2.3. RNA Probes Preparation and In Situ Hybridization

*ILK* sequence for RNA probes was obtained using primers listed in Supplementary Table S1 and with the method described elsewhere [33]. PCR was performed using Phusion™ High-Fidelity DNA Polymerase (Thermo Fisher Scientific, Warsaw, Poland) according to the manufacturer’s recommendation. PCR product was subsequently cloned into *EcoRI* (FastDigest, Thermo Fisher Scientific, Warsaw, Poland) cut pDrive plasmid (Qiagen, Wrocław, Poland) in the NEBuilder HiFi DNA Assembly Reaction (New England BioLabs, Ipswich, MA, USA). Finally, the obtained plasmid was sequenced. According to the manufacturer’s protocols, this construct acted as the template for preparing Digoxigenin-labeled RNA probes with DIG RNA Labeling Mix (Merck, Darmstadt, Germany) in an in vitro transcription reaction. Two types of riboprobes were prepared—antisense and sense (negative control) using T7 and SP6 RNA polymerase, respectively. In situ hybridization was carried out on chicken embryos at various stages fixed with 4% formaldehyde. In situ hybridization was carried out as described elsewhere [34]. Detection of *ILK* mRNA transcript was performed using digoxigenin-labeled antisense riboprobe, anti-Dig antibodies conjugated with alkaline phosphatase (AP), and its substrate-BCIP/NBT. For sectioning, embryos were embedded in 4% agarose, cut with McIlwain Tissue Chopper (Ted Pella, Inc., Redding, CA, USA), mounted on a microscopic glass with Glycergel Mounting Medium (Agilent Technologies, Inc., Santa Clara, CA, USA), and then imaged with fluorescence stereo microscope Leica M205 and Leica Application Suite (4.12.0).

### 2.4. Dorsal Root Ganglia Isolation, Dissociation, and Primary Culture

Fertilized chicken eggs were incubated at 37 °C and 80% humidity for 8 days. DRG were isolated from the trunk region of an 8-day old chick embryo based on protocols described elsewhere [35,36]. In the case of DRG single-cell culture, DRG were dissociated enzymatically by incubation in 0.25% trypsin/0.05% EDTA solution for 20 min and trituration through a small-bore pipette (20–200 µL) [36]. DRG explants or cells were cultured in N2 medium [98% DMEM/F-12 medium, 0.5 mg/mL bovine serum albumin (BSA), 2 mM l-glutamine, 1 × N-2 supplement, 100 ng/mL nerve growth factor (NGF)] [36]. DRG explants or cells were seeded on coated glass coverslips. The coverslips were coated first with poly-D-lysine (0.1 mg/mL) (Santa Cruz Biotechnology Inc., Heidelberg, Germany) for 1 h at room temperature, and after washing with sterile water, they were coated with laminin from Engelbreth-Holm-Swarm murine sarcoma basement membrane (EHS laminin, laminin-1) (Merck, Darmstadt, Germany) for 4 h in 37 °C. Laminin was used in the concentration of 1 µg/cm^2^ in Hanks’ Balanced Salt solution (HBSS). DRG were cultured at 37 °C in 5% CO_2_ for 48 h. Next, they were subjected to lysates collection or fixation for immunostaining.

### 2.5. Immunocytochemistry and Confocal Microscopy

DRG cultured on laminin-1-coated coverslips were washed with phosphate-buffered saline (PBS) and then fixed with 4% formaldehyde for 20 min. Blocking of non-specific bindings was performed by incubation in 1% bovine serum albumin (BSA) in PBS for 1 h. Non-permeabilized and permeabilized conditions were performed to visualize the intracellular and cell membrane localization of proteins. Immunocytochemical staining without permeabilization omitted the following 6 min incubation with 0.1% Triton X-100 in PBS. Additionally, all steps were performed at 4 °C, and no solution contained Triton-X-100. Coverslips were then incubated with primary antibodies overnight at 4 °C, washed with PBS, and incubated with secondary antibodies at room temperature for 1 h. The information about primary and secondary antibodies used for immunocytochemistry is shown in Supplementary Table S2. In non-permeabilized conditions, a mix of antibodies with lectin PHA-L from *Phaseolus vulgaris* was exceptionally diluted in Tris-buffered saline (TBS) with the addition of Ca^2+^ and Mn^2+^ instead of PBS. Alexa Fluor 350 conjugate phalloidin (Santa Cruz Biotechnology Inc., Warsaw, Poland) was used in 1:1000 dilution to detect filamentous actin. Hoechst (Thermo Fisher Scientific Inc., Warsaw, Poland) was used for the detection of the cell’s nucleus. Lectin PHA-L from *Phaseolus vulgaris* conjugated with Alexa Fluor 488 (Thermo Fisher Scientific Inc., Warsaw, Poland) was used in 1:100 dilution to control non-permeabilized conditions. PHA-L has already been used for cell surface staining [37]. At the end of the staining procedure, coverslips were mounted on glass slides using Dako Mounting Medium (Agilent Technologies, Inc., Santa Clara, CA, USA). Images were taken and analyzed with Leica TCS SP8 Confocal Laser Scanning Microscope and Leica Application Suite X (LAS X) software.

### 2.6. Western Blot Analysis

DRG explant cultures were lysed with urea buffer (50 mM Tris HCl pH 7.4, 5% SDS, 8.6% sucrose, 1 mM DTT, 0.45% urea) or CB (cytoplasmic proteins buffer) (10 mM Tris, pH 7.4, 100 mM NaCl, 1 mM EDTA, 1 mM EGTA, 1 mM NaF, 20 mM Na_4_P_2_O_7_, 2 mM Na_3_VO_4_, 1% Triton X-100, 10% glycerol, 0.1% SDS, 0.5% deoxycholate) supplemented with both PIC and phosphatase inhibitors cocktails 1:100 (Sigma-Aldrich, Poznań, Poland). Lysates were triply frozen and thawed and then centrifuged at 12,000× *g* for 5 min at 4 °C. Supernatants were stored at −80 °C until used for the analysis. The concentration of proteins was measured with the Pierce™ BCA Protein Assay Kit (Thermo Fisher Scientific Inc., Warsaw, Poland) or Bradford (Sigma-Aldrich, Poznań, Poland) assay according to the manufacturer’s instruction. DRG culture media were collected, centrifuged (1000× *g*, 10 min, 4 °C), and then concentrated with Amicon Ultra-4 Centrifugal Filter Unit (Millipore, Warsaw, Poland), and the concentration of proteins was measured with Bradford Reagent (Sigma-Aldrich, Poznań, Poland). The same amount of protein per sample (20 or 30 µg) was run on polyacrylamide gels by SDS-PAGE followed by transfer to nitrocellulose membrane [38]. Membranes were next blocked with 5% skimmed milk in Tris-buffered saline with 0.1% Tween 20 (TBS-T). A list of primary and secondary antibodies used for Western Blot analysis is shown in Supplementary Table S2. Proteins were visualized using Clarity Western ECL Substrate, ChemiDocTM MP System, and ImageLab 4.0 software, all from Bio-Rad (Hercules, CA, USA).

### 2.7. Antibodies-Mediated Disruption Of ILK Function

DRG were seeded on coverslips coated with poly-D-lysine (0.1 mg/mL) with the addition of 20 μg/mL MAPTRIX-L-IKVAV (Sigma-Aldrich, Poznań, Poland) or 10 μg/mL laminin-1. Alternatively, poly-D-lysine coated coverslips were coated with laminin-1 (1 µg/cm^2^). For disruption of ILK function, antibodies against ILK were added to the N2 medium in 10 μg/mL concentration. DRG cultured with the addition of normal rabbit in corresponding concentration were used as a control. DRG were cultured at 37 °C in 5% CO_2_ for 48 h. Then they were immunostained and subjected to further analyses such as DRG axonal outgrowth, the directionality of axons, SCPs migration, and growth cones’ morphology. Additionally, lysates were prepared from DRG upon the disruption of the ILK function assay.

### 2.8. Neurite Outgrowth Assay

Axonal outgrowth was measured based on the NF-m staining. For tracking individual neurites, the semi-automated Fiji application plugin [39] NeuriteJ [40] was used. The mean length of neurites was measured and then averaged for ten of the most extended neurites per DRG, as described elsewhere [37]. Results are presented as a mean length of neurites calculated based on nine DRG per condition.

### 2.9. The Directionality of DRG Neurite Extension

The directionality of extending neurites was calculated based on the images of DRG with detected mid-sized neurofilaments (NF-m). For each DRG, three areas starting from the edge of DRG body cell mass (BCM) were analyzed and averaged. Histograms’ data and color maps representing the neurites’ orientation were created with the ImageJ plugin, OrientationJ [41], based on settings described elsewhere [42]. Since the number of analyzed pixels differs between groups (images) due to the different numbers and lengths of axons, the neurites’ directionality analysis was performed based on areas under curves (AUC) calculations. |−44.5° to 44.5° AUC was divided by the sum of −90° to −45.5° and |45.5° to 90°. AUC. The higher number was obtained, the straighter neurites were. The histograms and raw data of (AUC) taken for the directionality analysis of extending DRG neurites are shown in Supplementary Table S3 and Figure S4. The results are reported as the normalized alignment of the AUC between the analyzed groups.

### 2.10. Migration of SCPs and the Length of Axonal Halo

Schwann cell precursors’ migration was measured based on DRG photos immunostained against Sox10. The axonal halo length was referred to NF-m staining. Both parameters were measured using the ImageJ plugin—Concentric Circles. The number of SCPs was determined with the ImageJ software by applying the function of “Particle analysis” with manual thresholding corresponding to the Sox10^+^ cells present out of BCM. Four images taken at different sides of the DRG were analyzed. The data were then averaged for each DRG. Schwann cell precursors’ migration and length of axonal halo were calculated for the ten most distant SCPs and neurites from the border of BCM. The results are presented as a mean SCPs distance and number, the mean length of axonal halo, and the ratio between SCPs and axonal halo length calculated based on means obtained for each DRG.

### 2.11. Analysis of Growth Cones’ Morphology

The length and the number of filopodia per growth cones were determined based on DRG photos stained with fluorescently labeled phalloidin. Ten growth cones per DRG were taken for analysis performed using plugin NeuriteJ [40] of ImageJ application. The data are presented as the mean length of filopodia and the number of filopodia per growth cone.

### 2.12. Experimental Design and Statistical Analysis

Experiments were performed using chicken *Gallus gallus domesticus* embryos (stage HH21 (E3.5), HH34 (E8), HH36 (E10)) according to the Hamburger and Hamilton classification. The data performed on DRG were obtained from at least nine DRG in three independently performed tests. Lysates of DRG cultures were analyzed from three independent sets of samples. All statistical analysis and graphing were carried out using GraphPad Prism 7 and 8 (GraphPad Software Inc., San Diego, CA, USA). First, the normality of distribution was determined using Shapiro–Wilks test and D’ Agostino and Pearson normality tests. The parametric and nonparametric versions of statistical tests were used for normal and abnormal distributed data sets, respectively. Statistical significances were calculated with two-tailed unpaired student’s *t*-test or ANOVA (one-way) with posthoc test (Dunnett’s multiple comparisons test), depending on data sets and experiments. Means ± standard deviations (SD) were used for the data reporting. The significance levels were defined as *p* < 0.05 (*), *p* < 0.01 (**), *p* < 0.001 (***), and *p* < 0.0001 (****).

## 3. Results

### 3.1. ILK Is Expressed in Chicken Embryo’s Dorsal Root Ganglia at mRNA and Protein Level, but It Is Not Secreted by DRG Cells

**Figure 1:**
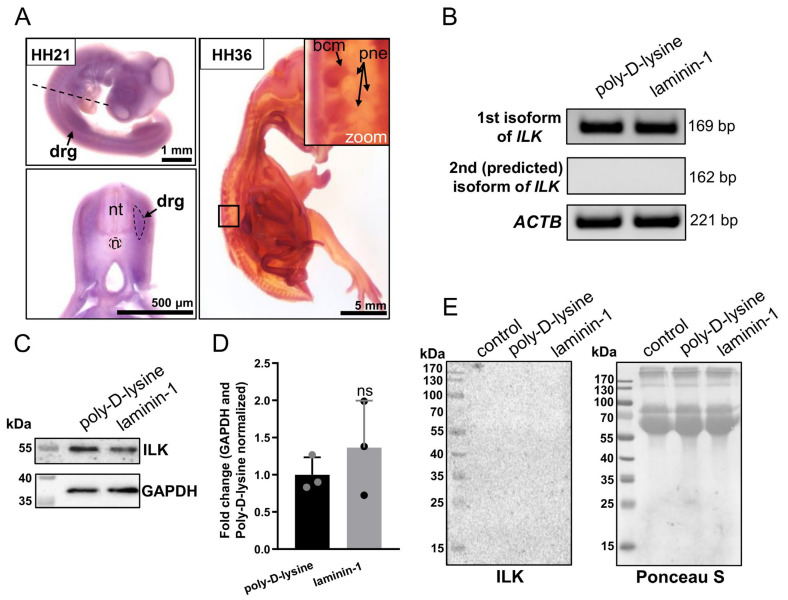
*ILK* is expressed in chicken embryo’s dorsal root ganglia at the mRNA and protein levels, but it is not secreted by DRG cells. (**A**) Analysis of the expression of *ILK* in the DRG of HH21 and HH36 stage chicken embryos using in situ hybridization assay. An antisense digoxigenin-labeled RNA probe against *ILK* mRNA transcript was used. The negative control performed using an RNA sense probe is presented in Supplementary Figure S1. The approximate schematic cutting line is highlighted with a black dashed line. Width of section—150 μm; bcm, body cell mass; drg, dorsal root ganglia; *n*, notochord; nt, neural tube; pne, peripheral nerves; (**B**) RT-PCR analysis of *ILK* expression in DRG isolated from E8 chicken embryo and then cultured on coverslips covered with poly-D-lysine and poly-D-lysine with laminin-1 for 48 h. PCRs were performed using primers (listed in Supplementary Table S1) specific for the transcript of first or second (predicted) ILK isoforms. *ACTB* transcript served here as control; (**C**) Western blot analysis of ILK level in DRG explants grown on coverslips coated with poly-D-lysine and laminin-1 for 48 h. Twenty micrograms of protein were loaded on every lane. Corresponding Ponceau S staining and whole membranes are shown in Supplementary Figure S2; (**D**) Densitometric analysis of ILK level in DRG cultured on coverslips coated with poly-D-lysine and laminin-1 for 48 h. Data were normalized to Ponceau S and GAPDH; (*n* = 3); (**E**) Western blot analysis of concentrated media obtained from DRG cultured on coverslips coated with poly-D-lysine and laminin-1 for 48 h. Thirty micrograms of protein were loaded on every lane. Ponceau S staining is shown as a control for protein loading.

To evaluate whether *ILK* is expressed in chicken embryo’s DRG, we performed in situ hybridization assays on chicken embryos at two developmental stages: HH21 (embryonic day E3.5) and HH36 (embryonic day E10). The HH36 stage represents a well-developed DRG. Digoxigenin-labeled antisense riboprobe recognizing *ILK* mRNA transcript, anti-digoxigenin antibodies conjugated with alkaline phosphatase (AP), and its substrate (BCIP/NBT solution) were used to detect *ILK*’s transcripts. We observed *ILK* expression in DRG at both the HH21 and HH36 stages (Figure 1A). Nevertheless, at stage HH36, we could more precisely determine which components of DRG express *ILK*. We noted the presence of *ILK* mRNA transcript in both the body cell mass of DRG and DRG’s peripheral nerves (Figure 1A). As shown in Figure 1, *ILK*, besides being expressed in DRG, is also expressed in multiple other organs and tissues. Chicken embryos hybridized with the *ILK* sense probe (control) are presented in Supplementary Figure S1. In the case of control embryos, we did not observe any specific positive signal (Supplementary Figure S1).

To confirm the expression of *ILK* in DRG at the mRNA level, we performed RT-PCR analysis on DRG cultured on poly-D-lysine or additionally laminin-1-coated surfaces for 48 h, as it has been reported that laminin-1 strongly stimulates the outgrowth of DRG peripheral nerves [12]. Based on the sequences found in the Pubmed gene database (gene ID: 374018), there may exist two *Gallus gallus domesticus* ILK isoforms, including one predicted ILK isoform. Therefore, we decided to verify the presence of transcripts separately for each isoform in DRG of chicken embryos using RT-PCR. The molecular mass of ILK isoform 1 is 59 kDa, and the predicted ILK isoform 2 is calculated as ca. 46 kDa. However, the insufficient difference between the nucleotide sequences of the transcripts for ILK isoforms made it impossible to construct RNA probes that specifically detected each isoform separately during in situ hybridization assay. Therefore, we conducted PCR using primer pairs specific to each isoform’s mRNA sequence (Supplementary Table S1). The data showed the presence of one type of mRNA for ILK isoform 1 (NCBI Reference Sequence: NM_204194.1) in DRG of the HH34-staged chicken embryo (Figure 1B). On the contrary, no product was detected for the transcript of the predicted ILK isoform 2 (XM_015280909.2). Moreover, we did not note any significant changes in the level of *ILK* expression between the control group and group stimulated to outgrowth by laminin-1.

Next, to determine the expression of ILK in chicken DRG, we performed Western blot analyses using antibodies against ILK and anti-GAPDH as control. Although ILK was expressed in DRG growing both on poly-D-lysine and on the laminin-1 coated surface (Figure 1C), the densitometric analysis showed no significant changes in the level of ILK expression between these groups (Figure 1D).

As we determined the expression of ILK in DRG, we wanted to verify whether ILK may be secreted by DRG cells. For this purpose, media from DRG were cultured for 48 h, then collected, concentrated, and subjected to Western blot analysis. We did not detect any ILK signal in the DRG culture media (Figure 1E). Ctrl-medium, obtained from wells without DRG, acted as a control and confirmed ILK’s absence in reagents used to formulate neuronal medium (N2). Ponceau S staining of membrane served as a loading control, as we did not find any proper protein marker that would be suitable for whole secreted protein determination. The lack of ILK in DRG medium allowed us to compare only Western blot results performed on DRG lysates to investigate the level of ILK.

To sum up, *ILK* is expressed in DRG of chicken embryos, but it is not secreted to the extracellular environment. There is only one ILK isoform in chicken DRG, and transcript and protein levels are not changed upon stimulation of DRG axons to extending process.

### 3.2. ILK Is Localized in the Soma and Neurites of DRG Neurons and SCPs

**Figure 2:**
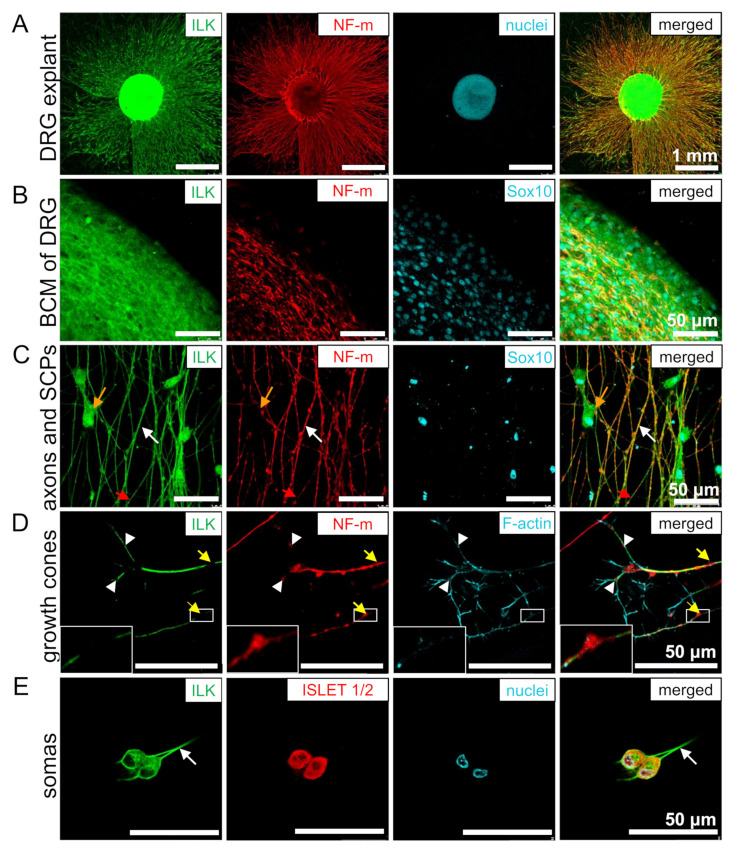
ILK is intracellularly localized in DRG neurons and SCPs. (**A**–**E**) Localization of integrin-linked kinase (ILK) in DRG explant and DRG single cells cultured on the surface coated with poly-D-lysine and additionally laminin-1. DRG explants and cells were cultured for 48 h and then immunostained in the permeabilized conditions with antibodies against ILK, NF-m, Sox10, and ISLET 1/2. To detect F-actin and cell nucleus, the specimens were stained with fluorescently labeled phalloidin and Hoechst 33342, respectively; (**A**) Images representing the whole DRG explant; (**B**) Images showing the presence of ILK in BCM of DRG; (**C**) Images representing ILK’s localization in axon shafts and SCPs. Orange arrows point to the presence of ILK in SCPs, white arrows indicate the localization of ILK in neurites, and the red arrows highlight neurofilament gaps in neurites; (**D**) Images showing ILK’s presence in growth cones and neurites. Yellow arrows point to the gaps in ILK-staining at the sites of neurofilaments accumulations. Enlarged images of these gaps are shown in the insets. White arrowheads highlight the filopodia of growth cones rich in NF-m; (**E**) The images of neuronal cell bodies were obtained from DRG single-cell cultures. White arrows point to axons.

To examine ILK’s localization in different cell types building DRG, they were isolated from HH34 chicken embryos and then grown as explants or dissociated cells for 48 h. DRG explants were grown on poly-D-lysine and laminin-1 coated glass coverslips. Immunostaining was performed on permeabilized cells. ILK was detected in the primary neurons of DRG, both in the cytoplasm and nucleus of neuronal cell’s bodies (somas), which can be observed as co-localization with Islet1/2 or as cells located in the body cell mass (BCM) (Figure 2A,B,E). ILK is also localized in the neurite shafts (Figure 2C, white arrows) and filopodia of growth cones (Figure 2D). This could be observed as a co-localization of ILK with NF-m and phalloidin, respectively. However, we noted that ILK did not localize in all formed filopodia, but perhaps only in those that became stable and mature axons after branching through growth cone bifurcation. This is supported by the observation that some NF-m signal was detectable in ILK-positive filopodia (Figure 2D, white arrowheads). Interestingly, ILK staining gaps were observed in the axons’ sites, where neurofilament accumulation occurs (highlighted with yellow arrows in Figure 2D). These gaps are probably mostly collateral branching points or nascent branches rich in actin patches. On the contrary, ILK is continued even along with the gaps of neurofilaments (Figure 2C, red arrows), which naturally occur during the rapid growth of neurites [43]. Additionally, ILK presence was noted in the cytoplasm and nucleus of spindle-shaped Sox10-positive cells (Figure 2D, orange arrows) migrating along DRG axons. Therefore, we identified these cells as Schwann cell precursors (SCPs).

Summarizing, ILK was localized in DRG neurons and SCPs.

### 3.3. ILK Localizes as well on the Surface of Somas and Neurites of DRG Neurons as well as SCPs

**Figure 3:**
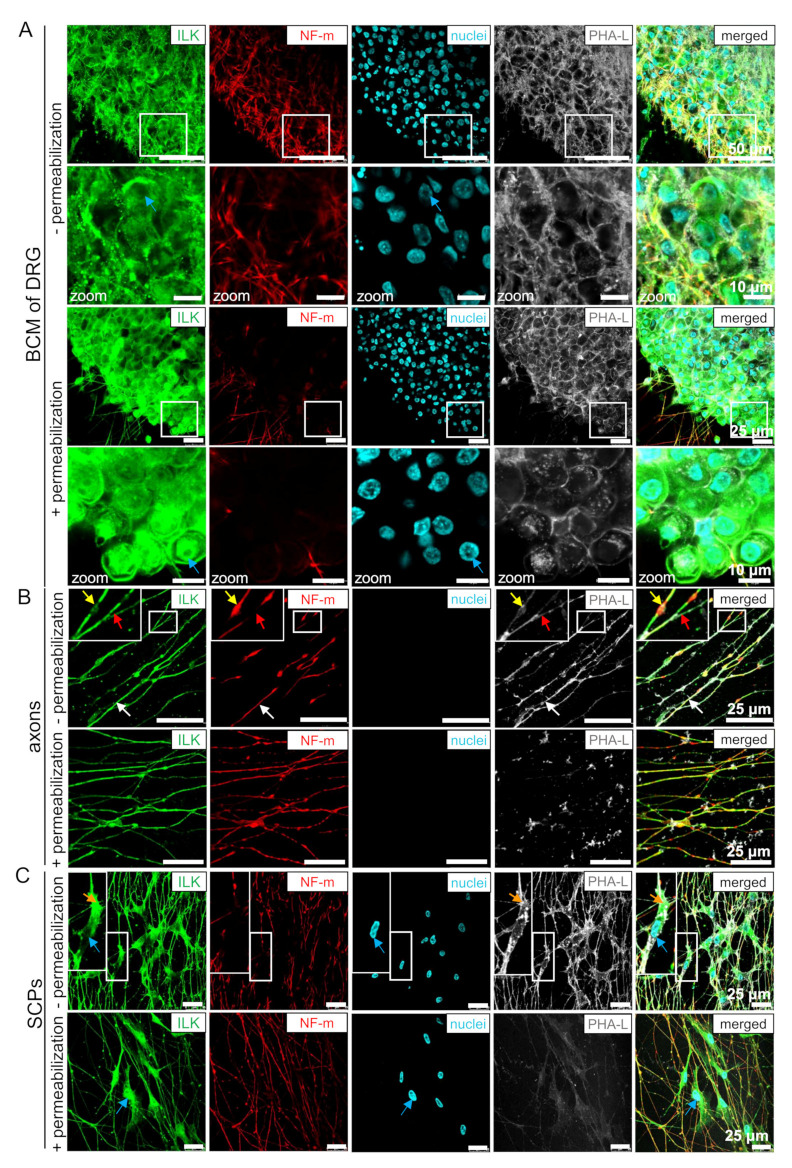
ILK is localized on the cell membrane of DRG neurons and SCPs. Localization of ILK on the cell membrane of DRG, which were cultured for 48 h on the surface coated with poly-D-lysine and additionally laminin-1. DRG explants were then stained and immunostained in the permeabilized and non-permeabilized conditions against ILK, NF-m, and Sox10. To detect the cell nucleus, the specimens were stained with Hoechst 33342. Lectin PHA-L conjugated with Alexa Fluor 488 was used for cell surface staining. A different pattern of PHA-L staining in permeabilized and non-permeabilized cells indicates the correctly performed immunostaining procedure. (**A**) The images show the localization of ILK on the surface of neuronal cell bodies (BCM). Blue arrows indicate cell nuclei; (**B**) The localization of ILK on the cell membrane of neurites. White arrows indicate the localization of ILK in neurites, yellow arrows point to the gaps in ILK-staining at the sites of neurofilaments accumulations, and the red arrows highlight neurofilament gaps in neurites; (**C**) Localization of ILK on the membrane of SCPs. Orange arrows indicate SCPs; blue arrows identify the cell nuclei. Enlarged images are shown in the insets or separate panels.

To evaluate whether ILK localizes on the membrane of DRG cells, we performed immunostaining in non-permeabilized conditions. DRG explants were additionally stained with *Phaseolus vulgaris* lectin L (PHA-L), which binds to tri/tetra-antennary N-glycans [44,45]. The localization of glycoproteins containing glycans on the cell surface allows binding of fluorescent PHA-L, giving a fluorescent signal on non-permeabilized neurites and SCPs (Figure 3). Permeabilization of the cell membrane leads to obliteration of PHA-L’s cell surface labeling. Thus, we did not note PHA-L presence in permeabilized neurites and SCPs (Figure 3B,C). However, PHA-L positive signal was observed in both permeabilized and non-permeabilized DRG somas, although it was more granular with thinner soma borders in permeabilized somas (Figure 2A). These differences result from PHA-L binding to glycoproteins located in vesicles of secretory and endocytic pathways of the permeabilized DRG somas, but still, they are evidence that the experiment was properly performed [46]. Based on this, we showed that ILK was localized on the membrane of DRG neuronal somas (Figure 3A), neurites (Figure 3B, white arrow), and SCPs (Figure 3C, orange arrow). Additionally, non-permeabilized neurons and SCPs, as opposed to permeabilized cells, did not exhibit ILK localization in the nuclei of these cells (Figure 3A,C; blue arrows). Relative to permeabilized cells, here we also observed ILK gaps or its less intensive staining on NF-m swellings (yellow arrows) but continuing ILK staining on NF-m pauses (red arrows) (Figure 3B). Additionally, NF-m staining is interrupted with longer pauses along DRG axons in comparison to the permeabilized conditions.

In conclusion, ILK localization on the cell membrane of DRG neurites, somas, and SCPs enables its exposure to ECM, facilitating their interactions.

### 3.4. Disruption of ILK’s Function Results in the Stimulation of Laminin-Mediated DRG Neurite Outgrowth In Vitro

Since ILK was presented on the membrane of DRG neurons, we decided to interrupt ILK function using anti-ILK IgG. It is common to block or activate membrane proteins exposed to ECM, such as receptors or integrins, using antibodies [47,48]. Then, we performed DRG neurite outgrowth assays to investigate ILK’s role in DRG axonal outgrowth in response to one of the laminin-mimicking pentapeptides, IKVAV (Ile-Lys-Val-Ala-Val), regulating the integrin-mediated neurite outgrowth and also in response to native laminin-1 [9]. DRG were cultured on poly-D-lysine with the addition of IKVAV or laminin-1 to the culture medium or cultured on laminin-1-coated glass coverslips. The laminin-1 coating surface is a commonly used method for the culture of neurons. However, we decided to use two types of laminin-1 administration methods for comparing the results with adding IKVAV to the medium. The anti-ILK antibodies were targeted to amino-acids residues 340–400 of the human ILK sequence. This means that the ILK region for antibodies binding overlapped with the integrin-binding region on the ILK structure, mapped between residues 293 and 451 [17]. The specificity of the anti-ILK antibodies is shown in Supplementary Figure S2A. Additionally, changes in ILK-activity upon ILK-disruption assay are shown in Supplementary Figure S3. We performed Western blot analyses of ILK-dependent proteins on DRG grown on laminin-1 and upon incubation with control-IgG and anti-ILK IgG. ILK can phosphorylate GSKβ (Ser 9), and Akt (Thr 308, Ser 473), making them markers commonly used for estimation of the kinase activity of ILK [49,50,51]. Vinculin, similarly to ILK, accumulates in focal adhesions, where it takes part in their formation and functioning [52]. We observed that the levels of pAkt (Thr 308), pAkt (Ser 473), and pGSKβ (Ser 9) were decreased upon the addition of anti-ILK antibodies to DRG cultured on laminin-1 (Supplementary Figure S3A,B). Intriguingly, the effect of the addition of control antibodies was prominent in the case of the level of active forms of Akt. This proves that the addition of control IgG does have effect on cellular processes. Thus, all of the obtained results upon ILK’s function modulation were compared to outcomes obtained upon addition of control IgG. The levels of Akt1/2/3, GSKβ, and vinculin were not altered. Corresponding Ponceau S-staining membranes and GAPDH level are shown in Supplementary Figure S3C.

**Figure 4:**
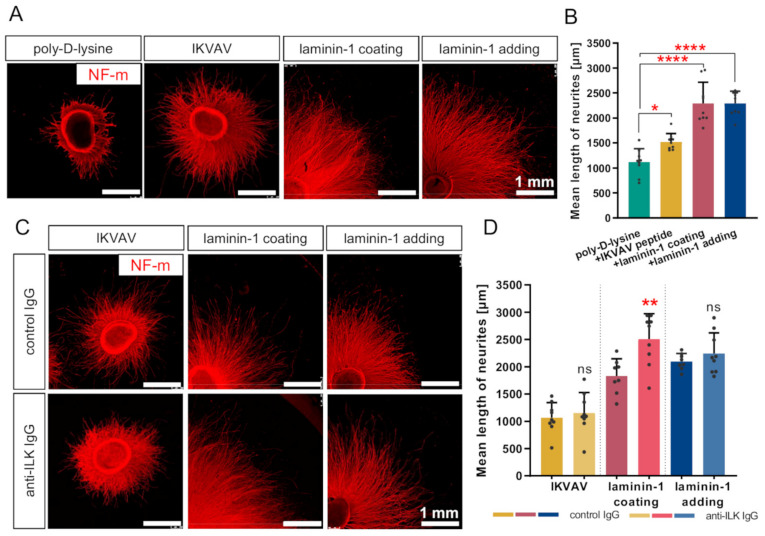
ILK plays a role in laminin-1-mediated DRG neurite outgrowth. DRG were cultured on a laminin-1-coated surface or with addition to a medium of IKVAV peptide or laminin-1 for 48 h. Separately, control or anti-ILK antibodies were added to the medium. Next, DRG were immunostained against NF-m to detect DRG neurites. (**A**) Representative images of DRG immunostained with anti-NF-m antibodies illustrating the DRG neurite outgrowth; (**B**) Quantification of DRG axonal outgrowth; *p* ≤ 0.05 (*); *p* ≤ 0.0001 (****); (*n* = 9); (**C**) Representative images of DRG immunostained against anti-NF-m, showing the DRG neurite outgrowth upon alteration of ILK’s function; (**D**) Quantification of DRG axonal outgrowth upon affecting of ILK’s action; *p* ≤ 0.01 (**); ns—not significant; (*n* = 9). Quantitative data (**B**,**D**) are presented as mean ± SD for ten the longest neurites per each of the nine DRG explants. Individual points represent averaged values obtained from the analysis of each DRG.

We confirmed previous findings showing that IKVAV could stimulate DRG axonal outgrowth compared to the poly-D-lysine (Figure 4A,B) [9]. Treatment of DRG with IKVAV resulted in longer neurites than the control group (poly-D-lysine) (1118 ± 265.3 vs. 1520 ± 167.2 μm). However, IKVAV did not exhibit the entire activity of laminin-1 at the concentration used. We also found that strong permissive properties of laminin-1 for DRG axonal elongation were observed regardless of how laminin-1 was administered during the experiment (coating glass coverslips or adding to culture medium) (Figure 4A,B). The mean length of neurites was similar in these two conditions (2291 ± 422.8 vs. 2292 ± 243.9 μm). DRG neurites stimulated with laminin-1 were approximately two times longer in comparison to the control group.

Additionally, we performed the ILK-function-disruption assay using anti-ILK antibodies added to the culture medium. Using anti-ILK antibodies, we could disrupt ILK function due to its localization on the DRG cell membrane (Figure 3), as it is not present in the culture medium (Figure 2F). DRG grown with the addition of normal rabbit IgG instead of anti-ILK antibodies were included as a control. The mean length of DRG axons grown with the addition of IKVAV or laminin-1 upon anti-ILK antibodies treatment did not change significantly compared to DRG grown with the addition of control antibodies (Figure 4C,D). Surprisingly, adding anti-ILK antibodies into DRG cultured on glass coverslips coated with laminin-1 instead of adding laminin to the medium resulted in statistically significant longer DRG neurites than the control group (1832 ± 315.5 vs. 2506 ± 466.4 μm) (Figure 4C,D).

In conclusion, IKVAV is a peptide responsible for laminin-1 stimulative properties for DRG neurite outgrowth in chicken. Affecting ILK’s function improved laminin-mediated stimulation of DRG neurites, but only in the condition of the laminin-1 coated surface. This indicates that the laminin-1 administration method is important for the impact of ILK on DRG axon outgrowth.

### 3.5. Anti-ILK Antibodies of High Specificity Impact the Directionality of Extending DRG Neurites In Vitro

**Figure 5:**
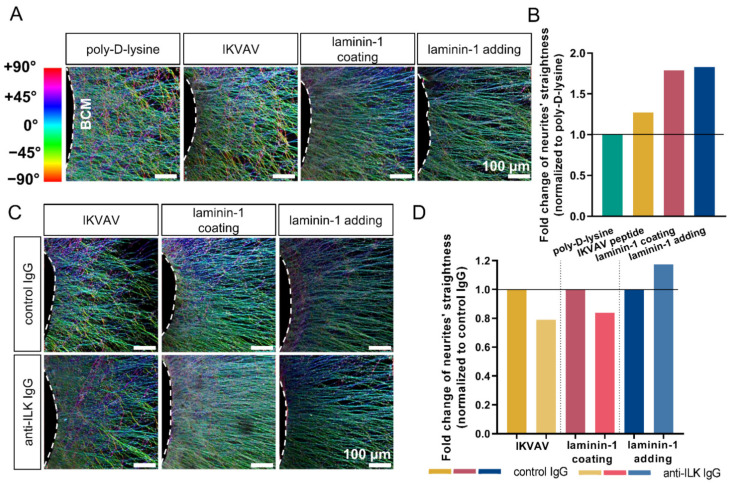
Interruption of ILK’s function results in changed directionality of DRG axons during extension. DRG were cultured on a laminin-1-coated surface, with IKVAV peptide, or with laminin-1 addition to the culture medium for 48 h. Additionally, control or anti-ILK antibodies were added to the medium. Next, DRG were immunostained against NF-m to visualize DRG neurites and analyzed using the OrientationJ plugin of the Fiji application. (**A**) Representative images showing color maps of DRG neurites’ orientation according to the color scale representing the deviation of the direction of neurites from 0° axis. White dashed lines represent the border of BCM; (**B**) Quantitative analysis of neurites’ straightness; (*n* = 9). (**C**) Representative images showing color maps of DRG upon treatment with anti-ILK antibodies; (**D**) Quantitative analysis of neurites’ straightness after treatment with anti-ILK antibodies; (*n* = 9). Final quantitative data are presented as fold changes of neurites’ straightness (normalized to the control groups for each test. A value higher than 1 indicates a more directed extension of DRG neurites, while values lower than 1 indicate their more chaotic extension. These data were calculated based on AUC from histograms generated by the OrientationJ plugin. More details can be found in the Materials and Methods section and in Supplementary Table S3 and Figure S4.

As directionality is an important aspect of DRG axonal extension, we decided to analyze the impact of ILK on DRG axonal outgrowth by studying whether adding anti-ILK antibodies impacts extending axons’ directionality. Using the OrientationJ plugin of ImageJ, we created color maps showing a visualization of neurites’ orientation (Figure 5A,C) and histograms (Supplementary Figure S4) used for quantitative analysis of data (AUC) (Supplementary Table S3). AUC for three ranges was analyzed: left |−90° to −45.5°|, middle |−44.5° to 44.5°| and right |45.5° to 90°|. Next, the middle AUC was divided by the sum of the left and right AUC. Obtained values (Supplementary Table S3) were normalized to the control groups and presented as fold change of neurites’ straightness (Figure 5B,D). The higher value obtained, the straighter neurites were, as there were more axons elongated in the direction of the |−44.5° to 44.5°| range.

We found that DRG cultured with the addition of IKVAV, laminin-1, or cultured on laminin-1 exhibited 27.5%, 78.9%, and 82.9% more straight axons in comparison to DRG grown only on poly-D-lysine, respectively (Figure 5B). This effect is visualized as color maps in Figure 5A. Relative to DRG neurite outgrowth, both methods of laminin administration have a comparable impact on axonal directionality.

The addition of anti-ILK antibodies decreased the straightness of DRG axons by about 20% and 16.2%, respectively, when cultured with the addition of IKVAV or on laminin-1 compared to the controls (Figure 5D). Intriguingly, DRG axons growing with the addition of laminin-1 and antibodies directed against ILK extended more systematically (about 17.5%) than the control group (Figure 5C,D).

To sum up, upon laminin-1 or IKVAV treatment, DRG resulted in a more ordered axon network. Anti-ILK antibodies impaired their straight elongation in the case of added to the medium laminin-1. On the contrary, when DRG were cultured on laminin-1 coating or in the presence of added IKVAV axons, elongation was straighter upon anti-ILK IgG addition. Thus, the directionality of axons depends on the method of substrate administration. Moreover, ILK’s effect on the directionality of DRG axons during extension also depends on the substrate type.

### 3.6. ILK Plays a Role in Laminin-Dependent Migration of Schwann Cell Precursors In Vitro, Increasing Their Number but Not Their Migration Distance

**Figure 6:**
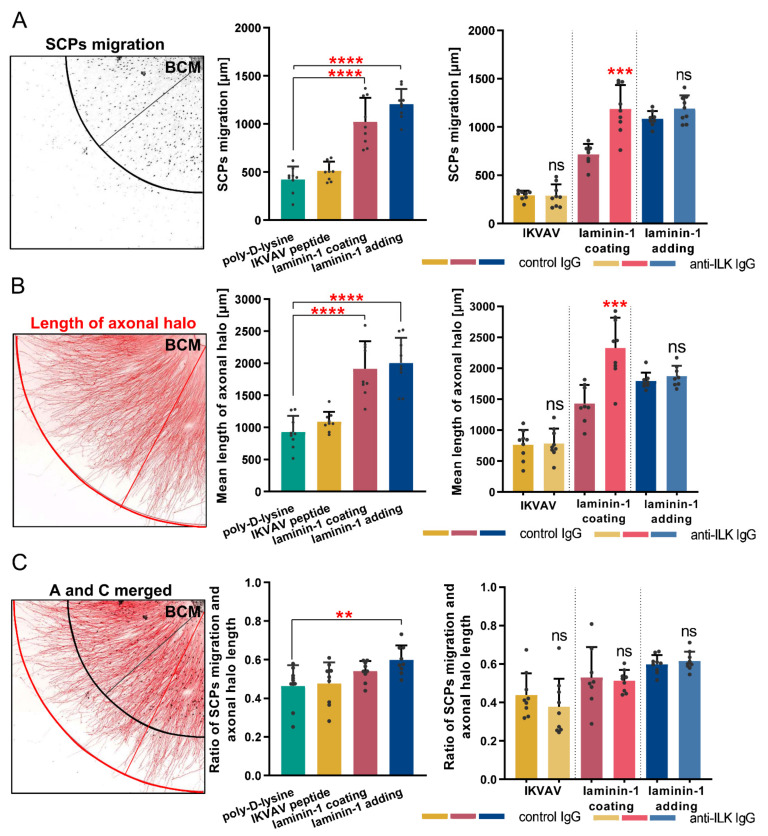
ILK does not play a role in laminin-1 mediated migration of Schwann cell precursors. DRG were cultured on a laminin-1-coated surface, with IKVAV peptide, or with laminin-1 for 48 h. Additionally, control or anti-ILK antibodies were added to the medium. Next, DRG were immunostained with anti-Sox10 and anti-NF-m antibodies to detect SCPs and DRG neurites, respectively. (**A**) The scheme of a part of DRG explant showing nuclei of SCPs and their distance of migration, and quantitative analysis of the impact of IKVAV and laminin-1 and disruption of the ILK’s function on the distance traveled by SCPs from the border of BCM; *p* ≤ 0.001 (***); *p* ≤ 0.0001 (****); ns—not significant; (*n* = 9); (**B**) The scheme of a part of DRG explant showing DRG axons and the length of their halo, and quantitative analysis of the impact of IKVAV and laminin-1 and disruption of the ILK’s function on the length of axonal halo measured from the border of BCM to the tips of the growth cones; *p* ≤ 0.001 (***); *p* ≤ 0.0001 (****); ns—not significant; (*n* = 9); (**C**) The scheme showing merged A and B schemes. Quantitative analysis of the impact of IKVAV and laminin-1 and disruption of the ILK function on the ratio between distance traveled by SCPs and length of DRG axonal halo; *p* ≤ 0.01 (**); ns—not significant; (*n* = 9). Four sides of each DRG were taken for analysis and then averaged. Individual points represent averaged values obtained from the analysis of each DRG. All bar charts show mean ± SD.

Next, we determined if the addition of anti-ILK IgG impacts SCPs’ migration, as they are present and support axons during extension. Since SCP migration depends on axonal signaling, we analyzed three different measurements: the mean distance traveled by SCPs, mean length of DRG axonal halo, and the ratio between these values, which is schematically presented in Figure 6A–C, respectively.

The quantitative analysis showed that laminin-1 (coated and added to medium) increased both SCPs’ migration and axonal halo length compared to the poly-D-lysine (Figure 6A,B). Nevertheless, we did not detect any statistically significant changes in the ratio between these values when DRG were cultured with the addition of IKVAV or on the laminin-1 coated surface (Figure 6C). The ratio of SCPs’ distance and axonal halo length was increased only when laminin-1 was added directly to the medium (Figure 6C). This indicates that the addition of laminin-1 to medium stimulated the migration of SCPs more than the axonal-mediated signals were able to.

On the contrary, anti-ILK IgG addition to DRG resulted in improved SCPs migration and axonal elongation when DRG were cultured on laminin-1 compared to the control IgG group (Figure 6A,B). However, the ratio between SCPs migration and axonal elongation was not altered (Figure 6C). No statistically significant changes in SCPs migration, the length of axonal halo, and the ratio of these values were observed upon addition of anti-ILK antibodies together with IKVAV or laminin-1, compared to control groups (Figure 6A–C).

To summarize, the addition of laminin-1 to DRG explant culture was responsible for increased SCPs migration distance. ILK did not impact SCPs migration distance along DRG axons in all tested conditions.

**Figure 7:**
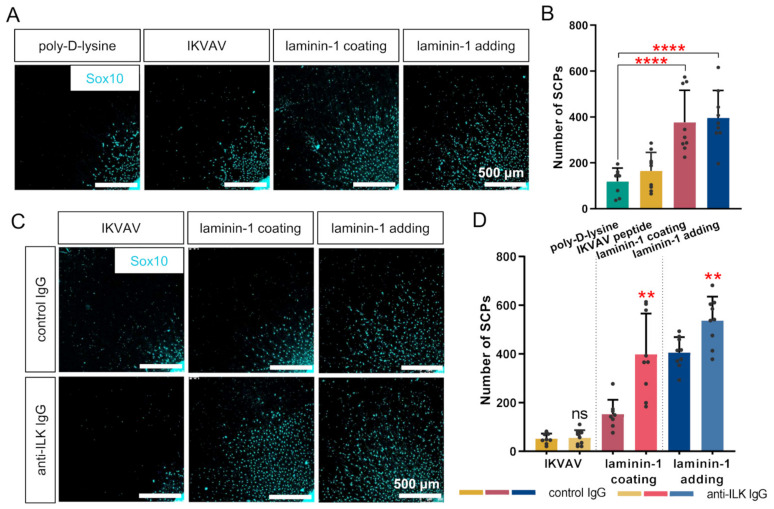
ILK is important for the number of SCPs migrating from DRG explant when cultured on laminin-1. DRG were cultured on a laminin-1-coated surface or with addition to the medium of IKVAV peptide or laminin-1 for 48 h. Additionally, control or anti-ILK antibodies were added to the medium. The number of SCPs was calculated based on immunostaining against Sox10. (**A**) Representative images showing the nuclei of SCPs present in DRG culture. (**B**) Quantitative analysis of the number of SCPs migrating along axons for DRG; *p* ≤ 0.0001 (****); (*n* = 9); (**C**) Images showing the nuclei of SCPs present in DRG culture after disrupting ILK’s function using anti-ILK antibodies; (**D**) Quantification of the number of SCPs migrating along axons for DRG cultured with antibodies against ILK; *p* ≤ 0.01 (**); ns—not significant; (*n* = 9). All quantitative data are presented as mean ± SD. The number of SCPs was measured for four sides of each analyzed DRG and then averaged. Individual points represent averaged values obtained from the analysis of each DRG.

Next, we decided to determine the number of migrating SCPs along axons. Quantitative analysis was made based on Sox10-immunostained cells identified as SCPs. Representative photos are presented in Figure 7A,C. The data demonstrate the similarly increased number of SCPs migrated from the BCM upon both laminin treatment methods (Figure 7B). However, the addition of IKVAV did not change the number of SCPs. Additional treatment of DRG with anti-ILK IgG resulted in a higher number of SCPs migrating along axons comparing to the control group (Figure 7D). We did not note any changes in DRG treated with IKVAV and anti-ILK antibodies compared to control (Figure 7D).

Summarizing, both laminin-1 treatment methods raised the number of migrating SCPs, which was additionally increased when anti-ILK antibodies were added to the DRG medium. The impact of ILK on the number of migrating SCPs was independent of the laminin-1 administration method.

### 3.7. Affecting ILK’s Function Increases the Number but Not the Length of Filopodia of DRG Neuronal Growth Cones In Vitro

**Figure 8:**
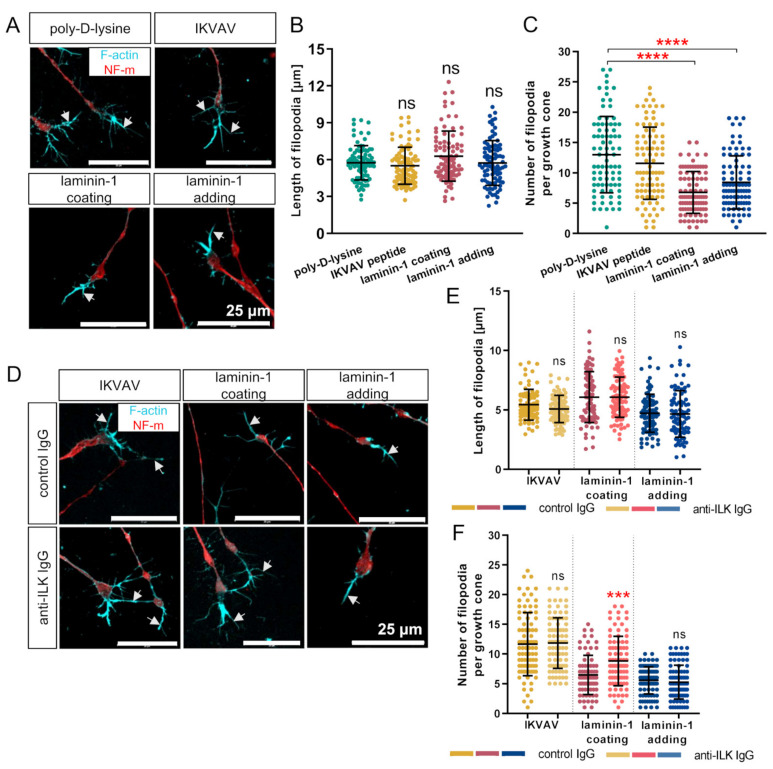
ILK modulates the morphology of a growth cone. DRG were cultured on a laminin-1-coated surface or with addition to the medium of IKVAV peptide or laminin-1 for 48 h. Additionally, control or anti-ILK antibodies were added to the medium. (**A**,**D**) DRG were then immunostained with NF-m to detect DRG neurites and stained with Alexa Fluor 568-phalloidin to label growth cones’ filopodia rich in F-actin. White arrows point at filopodia rich in F-actin. (**B**) Quantitative analysis of the length of filopodia of DRG growth cones; ns—not significant; (*n* = 90). (**C**) Quantitative analysis of the number of filopodia formed per one growth cone; *p* ≤ 0.0001 (****); (*n* = 90). (**E**) Quantification of the length of growth cone filopodia of DRG grown with the addition of anti-ILK antibodies; *p* ≤ 0.001 (***); ns—not significant; (*n* = 90). (**F**) Quantitative analysis of the number of filopodia formed per one growth cone after interruption of ILK’s function; *p* ≤ 0.001 (***); ns—not significant; (*n* = 90). All graphs show mean ± SD. The morphology of ten growth cones per DRG was analyzed.

Finally, we studied the morphology of axonal growth cones, as they are motile structures responding to axonal and extracellular environment signals. We visualized neuronal growth cones using antibodies directed against NF-m and fluorescently labeled phalloidin to detect filopodia (Figure 8A,D). Next, we calculated the mean number of filopodia per growth cone and measured the mean length of each formed filopodium.

We detected no statistically significant differences in the mean length of filopodia between all analyzed groups and upon anti-ILK antibodies administration (Figure 8B,E). On the contrary, the data exhibited a decreased number of filopodia per growth cone with both laminin treatment methods (6.76 ± 3.45 and 8.41 ± 4.41) in comparison to poly-D-lysine (12.99 ± 6.31) (Figure 8C). This was not observed in DRG cultured with the addition of IKVAV, as the mean number of filopodia per growth cone was 11.57 ± 5.96 (Figure 8C). Only DRG growing on laminin-1 upon anti-ILK antibodies treatment exhibited an increased number of filopodia per growth cone (8.83 ± 4.16) compared to control DRG (6.47 ± 3.3) (Figure 8F). The addition of anti-ILK antibodies to DRG cultured both with IKVAV or laminin-1 did not show any statistically significant differences compared to control IgG (Figure 8F).

In summary, only DRG stimulation with laminin-1 decreased the number of filopodia per growth cone, but it did not stimulate their extension. An impairment in ILK’s function resulted in a higher number of filopodia only in DRG grown on a laminin-1 coated surface.

## 4. Discussion

Integrin-mediated interactions between ECM and cells as a prerequisite for proper PNS development [53] and ILK’s association with integrins’ downstream signaling [17] inspired us to investigate whether ILK is involved in the formation of chicken dorsal root ganglia. No similar studies had been performed in chickens. Moreover, we have uncovered some novel aspects of ILK’s function in PNS development in mammals, such as the influence of ILK on SCPs migration from DRG or dependency of ILK function on the type of laminin-1 access. First, we determined the expression of *ILK* at mRNA and protein levels in DRG using in situ hybridization assay, RT-PCR, and Western blot analysis. However, the greater part of this paper is dedicated to experiments performed in vitro on isolated chicken DRG aimed at disrupting the function of ILK using anti-ILK antibodies. We decided to determine the impact of ILK on DRG development, especially in aspects that are directly correlated with the contact of cells with ECM via integrins, thus suggesting a possible role of ILK in these processes. We measured DRG neurite outgrowth, the directionality of DRG axons, SCPs migration, and filopodia morphology of neurites growth cones.

In advance of evaluating ILK’s role in DRG development, it was crucial to verify whether ILK is expressed in *Gallus gallus domesticus* DRG. Therefore, first, we proved the expression of *ILK* in the DRG of the chicken embryo at stages HH21 and HH36. Simultaneously, we report for the first time the presence of only one ILK isoform (isoform 1) in chicken DRG. In our opinion, this was an important point before further analysis, as it is already known that different isoforms of ILK partners, such as integrins or laminins, have different effects on axonal guidance and growth, allowing us to suppose that different ILK isoforms could also have different functional properties [11]. However, we found that the level of ILK in DRG explant cultured in the condition of axonal outgrowth stimulation (the presence of laminin-1) was not altered. Nevertheless, this does not exclude the possibility of changes in its activity, mirrored in ILK-dependent activation of Akt and GSK3β [54]. It is noteworthy that ILK’s kinase activity remains controversial [55].

Determining the protein localization in different cell compartments and structures can be very informative for evaluating their activities and role in the development of DRG. Previous work showed localization of ILK in rats hippocampal neuron tips [21] and its continual presence in mouse DRG neurites [19]. This is consistent with our results, showing the localization of ILK in chicken DRG neuronal bodies, neurites shafts, and growth cones. Although the literature reports the presence of ILK in neurons, we determined ILK’s localization in DRG for the first time under permeabilized and non-permeabilized conditions. Our data demonstrate both intracellular and cell membranous ILK localization in neuronal cell bodies, neurites, their growth cones, and SCPs. We report ILK’s presence on the surface of the cells and structures studied here. We are ensured that the non-permeabilization conditions were accurate as the staining with PHA-L lectin showed an altered staining pattern for detected tri/tetra-antennary N-glycans, which are predominantly localized on a cell surface [44]. ILK has no transmembrane domain. However, it possesses a phosphatidylinositol (3,4,5)-trisphosphate (PtdIns(3,4,5)*P*_3_) (PIP_3_) binding motive [56]. The binding of the pleckstrin homology (PH)-like domain present in ILK structure by PIP_3_ could participate in docking of ILK in the membrane [18,57]. ILK localizes in focal adhesions (FAs) sites, which are multi-protein structures connecting the cell with ECM and leading to actin remodeling, and thus cell migration and adhesion. It is conceivable that ILK could be docked to the cell membrane indirectly via binding to other members of FAs [58]. The localization of ILK has already been shown in the cell membrane of skeletal muscle cells [59]. The localization of ILK in nuclei of DRG neurons and SCP upon the permeabilized condition allows us to speculate this could be phosphorylated ILK, as previous studies indicated ILK phosphorylation essential for its nuclear localization [60,61]. The role of nuclear-localized ILK in proliferation and mitosis has already been reported [62,63]. Additionally, we found that ILK is not secreted by DRG neurons and SCPs, which had not been studied so far. In the literature, only one study is dedicated to ILK as a possible secreted protein, indicating the relationship between the level of ILK in exosomes with the inflammatory response of endothelial progenitor cell (EPC) [64]. As ILK is not secreted to culture medium, we assume that the anti-ILK IgG used in experiments impacted only the functionality of the ILK membranous fraction, which, because of its exposure to the ECM, might be crucial for the interaction of DRG cells with the extracellular environment and, therefore, their development. Alternatively, anti-ILK antibodies could be internalized (e.g., together with ILK’s binding partners, i.e., integrins). Integrin trafficking is a common mechanism found under normal and pathological conditions and is also important for axonal growth cones’ proper functioning [65]. Once antibodies against ILK are internalized they could act inside of a cell, influencing intracellular ILK’s function. These two possible scenarios should be examined in future work.

ECM proteins exhibit permissive, non-permissive, and inhibitory properties during PNS development dependent on the specificity of receptors localized on the cell’s surface [53,66]. Our data confirmed the stimulatory effect of laminin-1 on DRG neurite outgrowth, which is associated with the presence of laminins-specific integrins, i.e., β1 integrin with α1 or α3 subunit in DRG cells [67,68]. Here, we decided to evaluate the role of laminin-1 administered in two different forms (added to medium or coated) for the development of DRG, as the literature showed that the development of PNS strongly depends on ECM proteins coming from different sources in vivo. ECM proteins can act there as the immobilized substrates (networks, sheets or basement membrane) for migrating cells and elongating axons [53]. However, ECM proteins, such as laminins, can also be secreted by various cells, e.g., muscles, neural and endothelial cells, neuroretina, or Schwann cells, all in a tissue-specific manner [69,70]. Schwann cells can secrete and deposit laminin in ECM, which is essential for the radial sorting of axons and the myelination process [69]. We present here laminin-1 stimulation of DRG neurite elongation, being independent of the laminin-1 administration method. Additionally, we confirmed that a laminin pentapeptide, IKVAV, mimicking laminin, also stimulates axonal outgrowth [9], but does not exhibit full laminin-1 properties, at least not for chicken tissue. Next, we determined the role of ILK in laminin-1- and IKVAV-mediated DRG axonal outgrowth using anti-ILK IgG. Others have shown that ILK overexpression resulted in the activation of Akt and inhibition of GSK, seen as the increased level of their phosphorylated forms on Ser 473 and Ser 9, respectively [18,71]. Inhibition of ILK caused a reduction in the level of pAkt (Ser 473) and pGSKβ (Ser 9) in hippocampal neurons [21]. Loss of ILK was also found to decrease the phosphorylation of Akt on Ser 473, and its increase on Thr 308 in mouse sciatic nerves [22]. On the contrary, an unchanged level of Akt phosphorylation at Thr 308 was reported, when the *ILK* expression was silenced in colon cancer cells [72]. It proves that the decreased level of Akt phosphorylation on Ser 473 correlates with decreased ILK-activity, nevertheless the impact of ILK on Akt phosphorylation on Thr 308 depends on a tissue context. We showed here that the treatment of cultured on laminin-1 DRG with anti-ILK IgG caused the changes in the level of proteins, being part of major ILK downstream signaling pathways. We assume that the decreased level of pGSKβ (Ser 9), pAkt (Thr 308), and pAkt (Ser 473) in comparison to total forms may indicate that used here anti-ILK IgG somewhat inhibit than increase the activity of ILK. Additionally, this conclusion is supported by the unaltered vinculin level upon treatment of DRG with anti-ILK IgG. An unaltered level of vinculin and its localization was shown upon ILK’s inhibition [73]. It is crucial to note here that upon control IgG administration we observed the dramatic decrease in the level of Akt phosphorylated on Ser 473, what implies that treating the DRG with control antibodies affects some of their features. For instance, that was seen in the case of assessment of the length of extending axons. Addition of control IgG in the presence of laminin resulted in shorter axons than for untreated DRG. Thus, it shows that comparing the results obtained upon anti-ILK IgG administration to the outcomes obtained after addition of control IgG was correct. This situation is similar to the analysis of experiments performed on transfected cells. Transfection itself influences cells, thus it is crucial to compare obtained results with those obtained for cells transfected with the control vector.

Intriguingly, the response to the interruption of ILK’s function was dependent on the type of laminin-1 treatment. Others have shown that soluble laminin polymers, structurally different at various pH, enhance axon outgrowth, depending on integrin’s subunits [74]. On the other hand, laminin-1-coating forms aggregates consisting of a matrix or flat network [75]. This can reflect the in vivo state during PNS development where laminin-1, being the substrate or secreted protein, might exist in different structural forms, the synergy/cooperation between these two forms may be responsible for the proper development/functioning of PNS. Therefore, we believe that promoted DRG axonal elongation on laminin-1 upon anti-ILK IgG treatment could be caused by a laminin-1 different structure, allowing ILK-associated integrins access to it. However, it may also be associated with neuron-ECM adhesion changes since the adhesive substratum properties strongly correlate with neurite elongation [76]. Alternatively, different mechanisms may exist when DRG cells attach to the laminin-1-coated surface and when they bind to laminin-1 present in the medium. Surprisingly, ILK’s role in DRG axonal outgrowth was not IKVAV-mediated. The impact of ILK on IKVAV-independent but laminin-1-dependent DRG neurite outgrowth allows speculation about the existence of another laminin-1 peptide responsible for laminin-1 impact on axonal outgrowth. Another explanation might be the fact that even if ILK could be important for IKVAV-mediated axonal outgrowth, this would only be possible when IKVAV acted as native laminin, since IKVAV could be conformationally differently arranged and thus be more available for ILK-associated integrins. That certainly requires further studies.

The growth cones are motile structures located at the tips of neurons. They interact with ECM, thereby navigating and extending neurites [77]. Their morphological changes, depending on ECM, include actin remodeling, which accumulates in growth cones’ filopodia and lamellipodia in the filamentous form [78]. We show that IKVAV was unrelated to the protrusion and retraction of growth cones. However, reduced filopodia number was the only morphological change after laminin-1 stimulation. This might indicate an inverse correlation between the filopodia number and DRG neurite outgrowth. Others also reported a negative correlation between growth cone size and neurite outgrowth but using *Aplysia* bag cell neurons [79]. Simultaneously, it has been shown that inhibited growth cones are smaller with no or a low number of filopodia, which are additionally shorter due to the decreased actin polymerization. Larger and more complex growth cones (increased actin polymerization) are classified as stimulated to move [80,81]. We hypothesize that DRG exhibited rapid growth and the most extended neurites after 48 h of laminin-1 treatment, but they stopped being stimulated and could not extend anymore. It was observed, therefore, as shorter filopodia. Intriguingly, DRG growing on laminin-1 in the presence of anti-ILK antibodies exhibited longer DRG neurites and a higher number of filopodia. We assume that they were continuously stimulated after disrupting ILK’s function, and they retained their extension properties. This could be the result of ILK-mediated actin remodeling in growth cones.

The proper directionality of outgrowing axons, dependent on the substrate stiffness, is essential for axonal development, growth, and regeneration [82]. However, disturbances in axon directionality occur during both the proper development and in disorders, like Alzheimer’s disease [83,84]. We conclude here that laminin-1 is essential for the directionality of axonal outgrowth via IKVAV, but the administration method of laminin-1 is irrelevant. Intriguingly, it was conditional for ILK-mediated impact on axonal orientation. Anti-ILK antibodies caused decreased straightness of DRG axons cultured with the addition of IKVAV or laminin-1. In contrast, increased straightness was observed in DRG grown on laminin-1. Nevertheless, the mechanism responsible for impaired directionality upon anti-ILK antibody treatment is unknown. We think that this might be connected with changes in, e.g., axonal branching, fasciculation, or navigation issues during extension.

SCPs actively migrate and proliferate along peripheral nerves to give rise to Schwann cells and melanocytes [3]. Their migrative, proliferative, and spreading properties are partly dependent on axon-derived signals [85]. Our studies demonstrate laminin-1 involvement in SCPs migration from the BCM. Although both laminin-1 administration methods exhibited improved distances and number of SCPs, axon-derived signals were probably responsible for that. The higher ratio between axonal halo length and SCPs migration, observed upon laminin-1 addition to medium, was caused by laminin-1. Increased SCPs migration upon anti-ILK antibodies treatment of DRG grown on laminin-1 was caused by promoted neurite elongation, which then influences SCPs. As the ratio of SCPs migration and axon halo length was not changed, still the number of SCPs was increased, which indicates that their emigration from BCM was altered upon anti-ILK antibodies, but the migration along axons was not. This might also be connected with SCPs differentiation to immature Schwann cells (SCs) as they usually exhibited increased proliferation [86]. Since ILK was localized both in SCPs and neurons, we cannot conclude that interruption of ILK function using anti-ILK antibodies impacted directly on DRG neurite outgrowth, and the changes of SCPs number were the result of that, or whether ILK impacted SCPs what consequently made changes it in axonal elongation. The influence of ILK on the number of SCPs is very intriguing and worth deciphering. As others have shown ILK’s role in SCs radial sorting of axons [22], we show that ILK is essential for SCPs involvement in the chicken DRG development.

## 5. Conclusions

Although ILK is extensively studied, the researchers mainly focus on the link between ILK functions and different types of tumors, as understanding its role may lead to the development of new therapies. The role of ILK in proper functioning and development, especially the nervous system, is also the subject of many studies. However, most of them are focused on the central nervous system (CNS). Only a few studies show whether and in what manner ILK affects the development of the peripheral nervous system and its proper functioning. It is worth remembering that diseases of the peripheral nervous system, such as neurofibromatosis, peripheral neuropathy, amyotrophic lateral sclerosis, and neuroblastoma, are quite common and worth studying. Based on the novel results presented here, we conclude that ILK, via its interactions with an ECM compound-laminin-1, is involved in the PNS development, modulating axonal outgrowth, sensory neuron morphology, and SCPs number. We believe that our results provide a sound basis for future research investigating the mechanisms responsible for these actions, leading to a better understanding of PNS-origin diseases and ILK’s role in them. We think that it would be beneficial as well to compare these results with outcomes from studies on in ovo model. For this purpose, in our future studies we plan to perform silencing of *ILK* expression using shRNA and in ovo electroporation technique.

## Data Availability

Not applicable.

## Supplementary Materials

The following are available online at https://www.mdpi.com/article/10.3390/cells10071666/s1, Figure S1: In situ hybridization results showing the negative control for HH21-staged chicken embryo, Figure S2: The whole membrane of Western Blot analysis shown in Figure 1C, Figure S3: The verification of ILK activity, presented as the level of ILK-dependent proteins upon ILK-alteration assay, Figure S4: Histograms showing the distribution of DRG axons’ orientation, Table S1: List of primers with their sequences and melting temperatures (Tm) used for RT-PCR, Table S2: List of antibodies used in the study, Table S3: Areas under curve data from the analysis of the neurites’ directionality.

## Abbreviations

AUC	Area under the curve
BCM	Body cell mass
DRG	Dorsal root ganglion/ganglia
ECM	Extracellular matrix
F-actin	Filamentous actin
HH stage	chicken developmental stage according to Hamburger and Hamilton
ILK	Integrin-linked kinase
IgG	Immunoglobulin G
NF-m	Mid-sized neurofilaments
NGF	Nerve growth factor
PNS	Peripheral nervous system
SCs	Schwann cells
SCPs	Schwann cell precursors
