# Supplementary material for: Integrin-Linked Kinase (ILK) Plays an Important Role in the Laminin-Dependent Development of Dorsal Root Ganglia during Chicken Embryogenesis
Source: Cells. 2021 Jul 2;10(7):1666. doi: 10.3390/cells10071666 (PMC8304069; doi:10.3390/cells10071666)
Supplement: Supplementary file 1 [file cells-10-01666-s001.zip › cells-1257362-supplementary.pptx]

## Slide 1
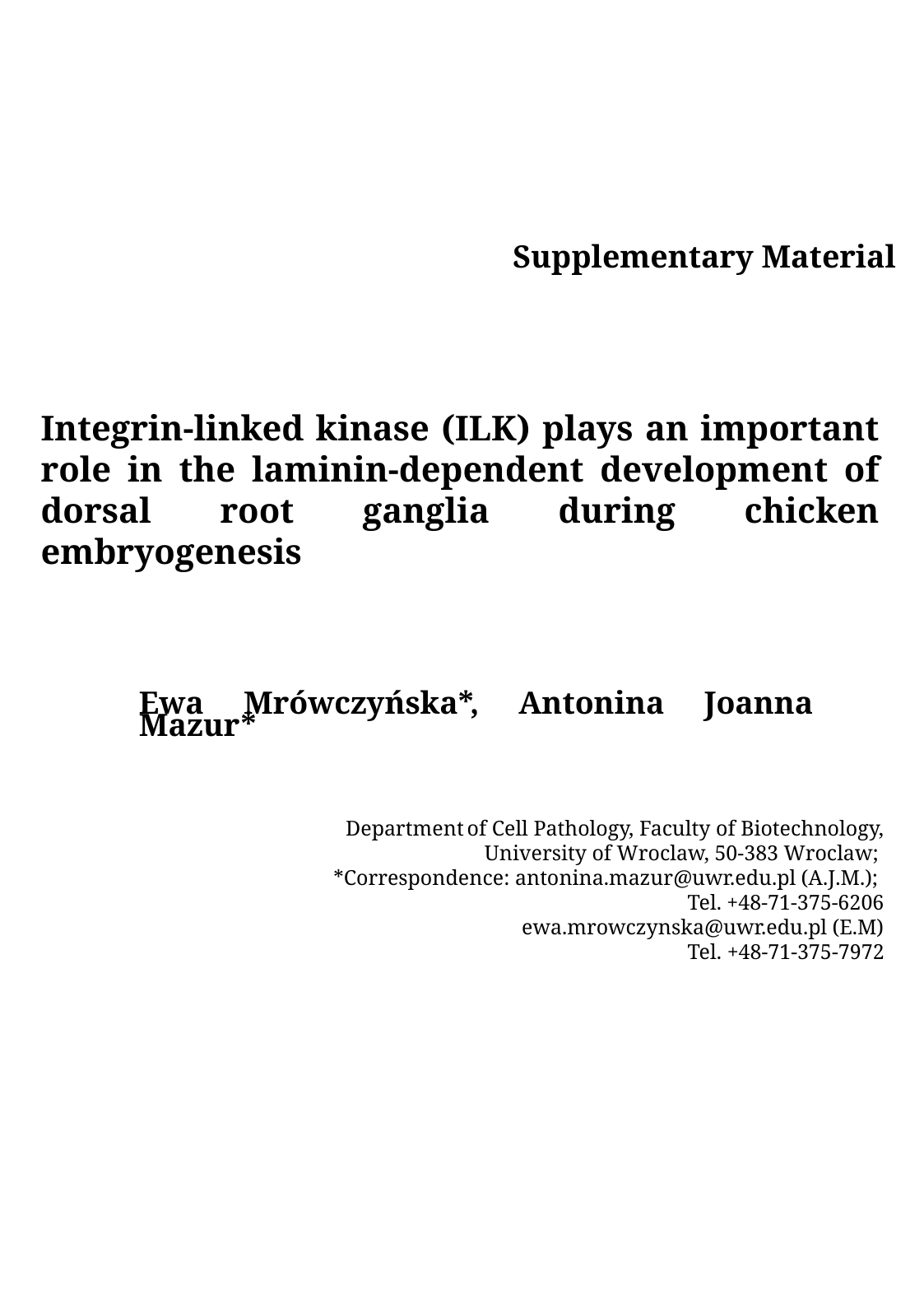

Supplementary Material
Integrin-linked kinase (ILK) plays an important role in the laminin-dependent development of dorsal root ganglia during chicken embryogenesis
Ewa Mrówczyńska*, Antonina Joanna Mazur*
Department of Cell Pathology, Faculty of Biotechnology, University of Wroclaw, 50-383 Wroclaw;
*Correspondence: antonina.mazur@uwr.edu.pl (A.J.M.);
Tel. +48-71-375-6206
ewa.mrowczynska@uwr.edu.pl (E.M)
Tel. +48-71-375-7972

## Slide 2
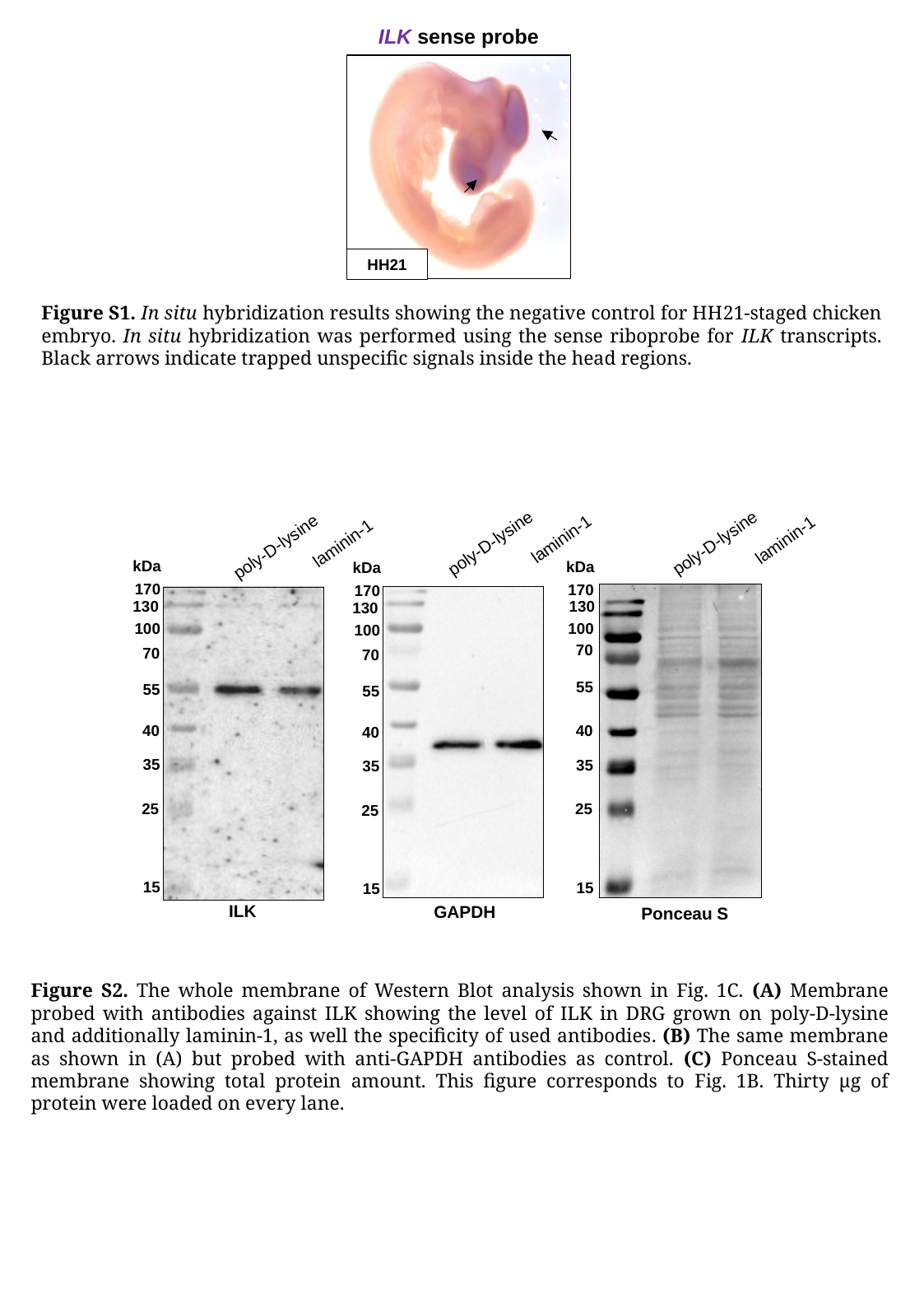

ILK sense probe
HH21
Figure S1. In situ hybridization results showing the negative control for HH21-staged chicken embryo. In situ hybridization was performed using the sense riboprobe for ILK transcripts. Black arrows indicate trapped unspecific signals inside the head regions.
laminin-1
laminin-1
poly-D-lysine
laminin-1
poly-D-lysine
poly-D-lysine
kDa
kDa
kDa
170
170
170
130
130
130
100
100
100
70
70
70
55
55
55
40
40
40
35
35
35
25
25
25
15
15
15
ILK
GAPDH
Ponceau S
Figure S2. The whole membrane of Western Blot analysis shown in Fig. 1C. (A) Membrane probed with antibodies against ILK showing the level of ILK in DRG grown on poly-D-lysine and additionally laminin-1, as well the specificity of used antibodies. (B) The same membrane as shown in (A) but probed with anti-GAPDH antibodies as control. (C) Ponceau S-stained membrane showing total protein amount. This figure corresponds to Fig. 1B. Thirty μg of protein were loaded on every lane.

## Slide 3
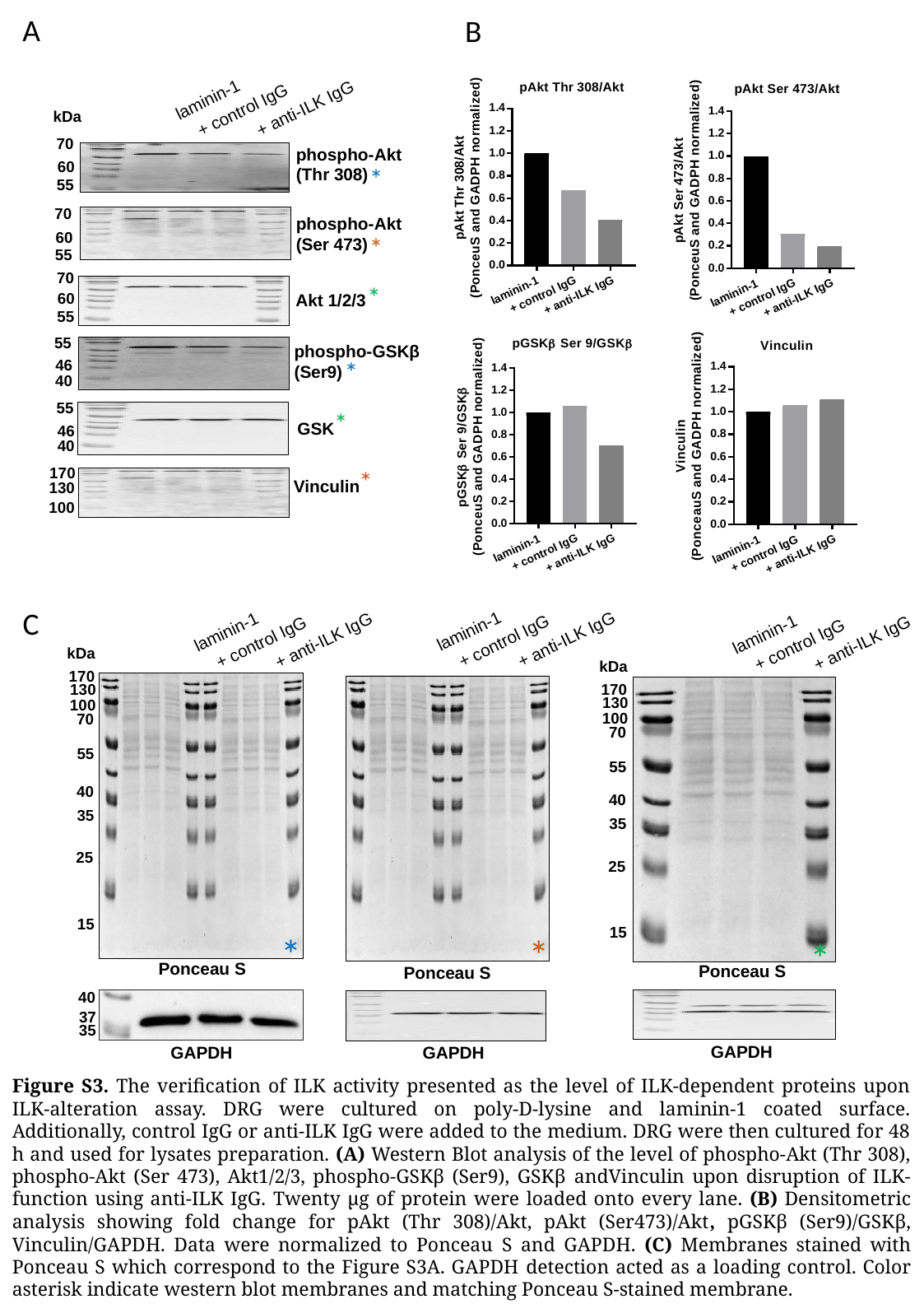

A
B
laminin-1
+ anti-ILK IgG
+ control IgG
kDa
70
phospho-Akt
(Thr 308)
60
*
55
70
phospho-Akt
(Ser 473)
60
*
55
70
*
60
Akt 1/2/3
55
55
phospho-GSKβ
(Ser9)
*
46
40
55
*
GSK
46
40
170
*
Vinculin
130
100
C
laminin-1
laminin-1
laminin-1
+ anti-ILK IgG
+ anti-ILK IgG
+ control IgG
+ control IgG
+ anti-ILK IgG
+ control IgG
kDa
kDa
170
130
170
130
100
100
70
70
55
55
40
40
35
35
25
25
15
15
*
*
*
*
Ponceau S
Ponceau S
Ponceau S
40
37
35
GAPDH
GAPDH
GAPDH
Figure S3. The verification of ILK activity presented as the level of ILK-dependent proteins upon ILK-alteration assay. DRG were cultured on poly-D-lysine and laminin-1 coated surface. Additionally, control IgG or anti-ILK IgG were added to the medium. DRG were then cultured for 48 h and used for lysates preparation. (A) Western Blot analysis of the level of phospho-Akt (Thr 308), phospho-Akt (Ser 473), Akt1/2/3, phospho-GSKβ (Ser9), GSKβ andVinculin upon disruption of ILK-function using anti-ILK IgG. Twenty μg of protein were loaded onto every lane. (B) Densitometric analysis showing fold change for pAkt (Thr 308)/Akt, pAkt (Ser473)/Akt, pGSKβ (Ser9)/GSKβ, Vinculin/GAPDH. Data were normalized to Ponceau S and GAPDH. (C) Membranes stained with Ponceau S which correspond to the Figure S3A. GAPDH detection acted as a loading control. Color asterisk indicate western blot membranes and matching Ponceau S-stained membrane.

## Slide 4
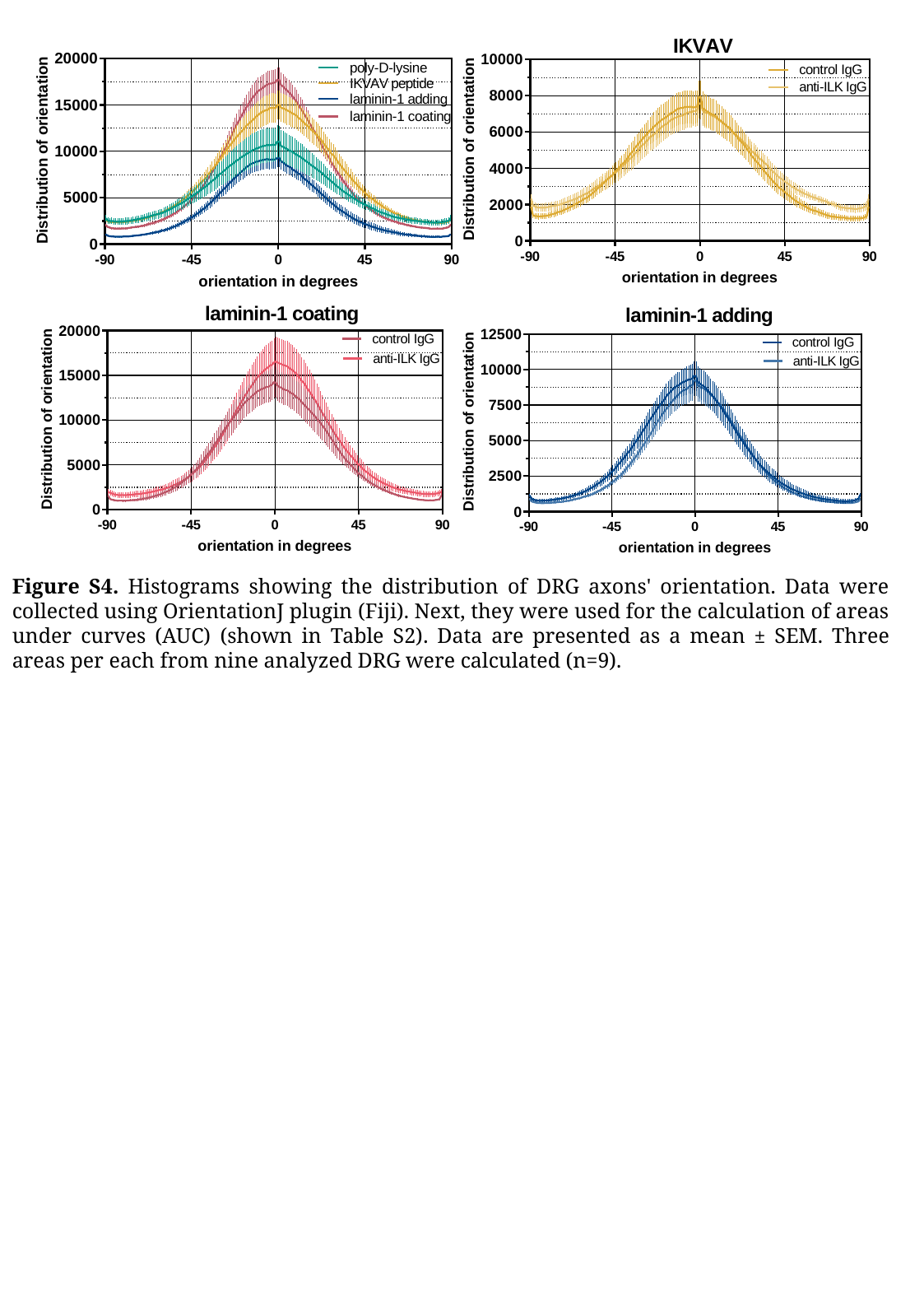

Figure S4. Histograms showing the distribution of DRG axons' orientation. Data were collected using OrientationJ plugin (Fiji). Next, they were used for the calculation of areas under curves (AUC) (shown in Table S2). Data are presented as a mean ± SEM. Three areas per each from nine analyzed DRG were calculated (n=9).

## Slide 5
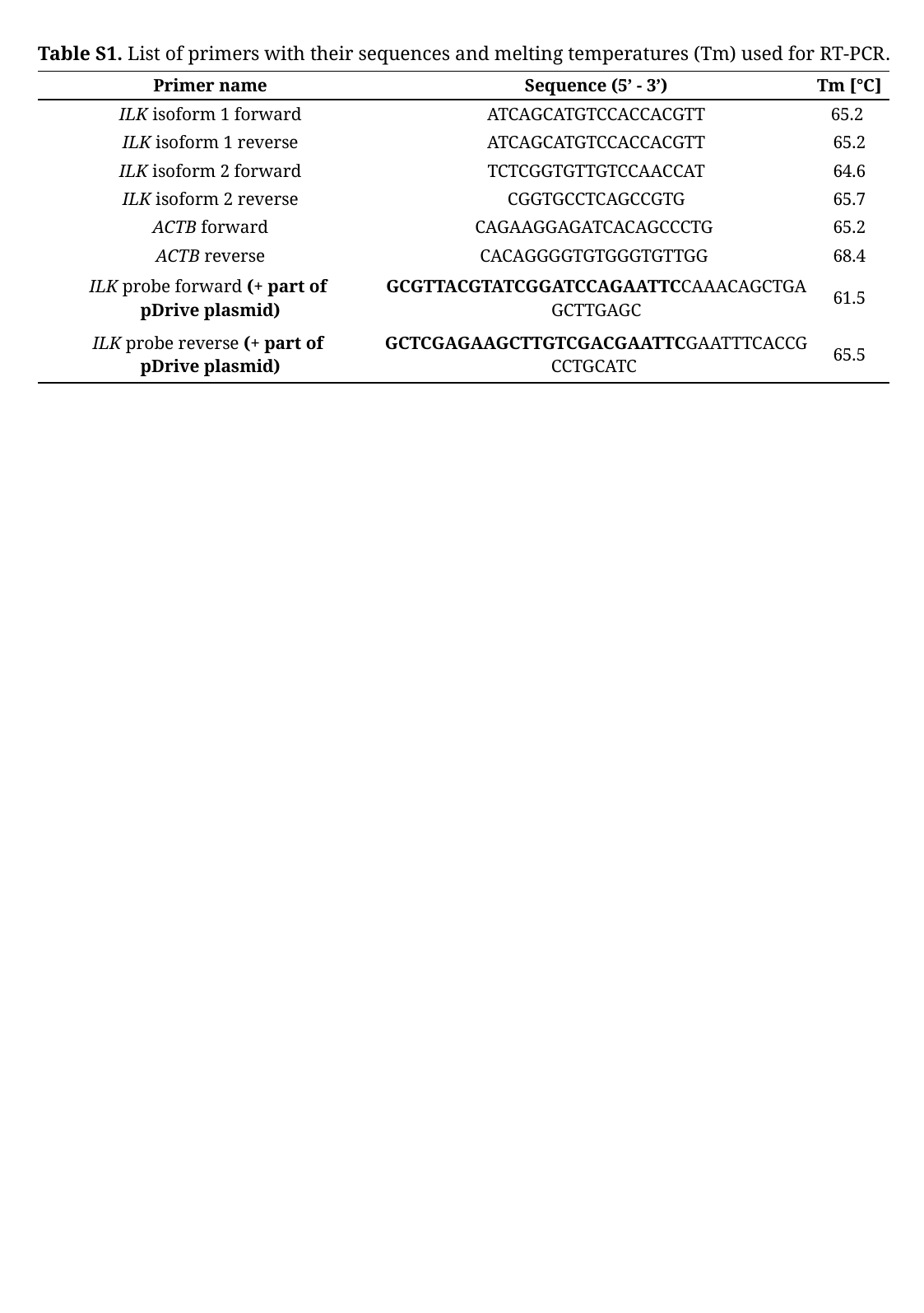

Table S1. List of primers with their sequences and melting temperatures (Tm) used for RT-PCR.
| Primer name | Sequence (5’ - 3’) | Tm [°C] |
| --- | --- | --- |
| ILK isoform 1 forward | ATCAGCATGTCCACCACGTT | 65.2 |
| ILK isoform 1 reverse | ATCAGCATGTCCACCACGTT | 65.2 |
| ILK isoform 2 forward | TCTCGGTGTTGTCCAACCAT | 64.6 |
| ILK isoform 2 reverse | CGGTGCCTCAGCCGTG | 65.7 |
| ACTB forward | CAGAAGGAGATCACAGCCCTG | 65.2 |
| ACTB reverse | CACAGGGGTGTGGGTGTTGG | 68.4 |
| ILK probe forward (+ part of pDrive plasmid) | GCGTTACGTATCGGATCCAGAATTCCAAACAGCTGAGCTTGAGC | 61.5 |
| ILK probe reverse (+ part of pDrive plasmid) | GCTCGAGAAGCTTGTCGACGAATTCGAATTTCACCGCCTGCATC | 65.5 |

## Slide 6
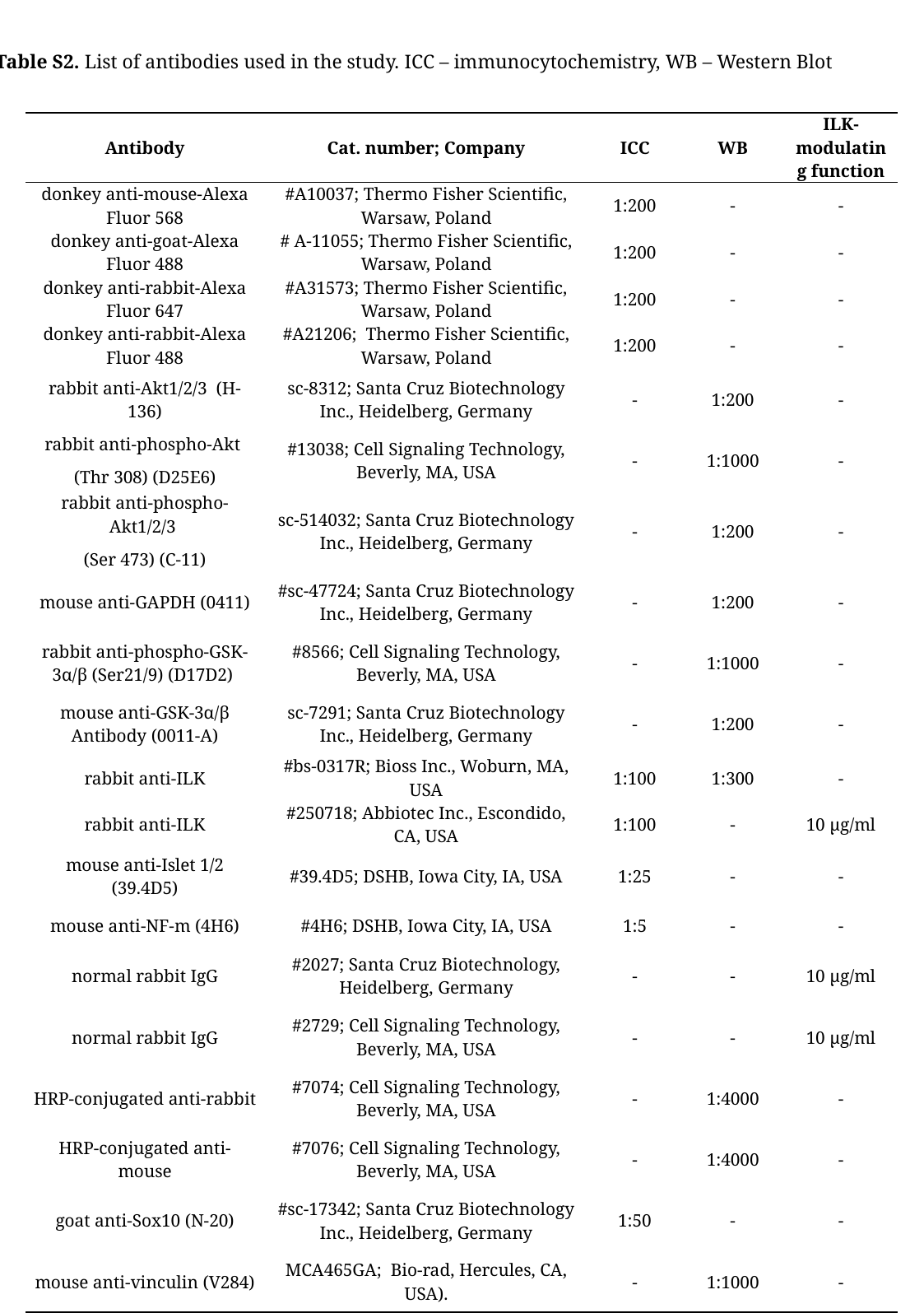

Table S2. List of antibodies used in the study. ICC – immunocytochemistry, WB – Western Blot
| Antibody | Cat. number; Company | ICC | WB | ILK- modulating function |
| --- | --- | --- | --- | --- |
| donkey anti-mouse-Alexa Fluor 568 | #A10037; Thermo Fisher Scientific, Warsaw, Poland | 1:200 | - | - |
| donkey anti-goat-Alexa Fluor 488 | # A-11055; Thermo Fisher Scientific, Warsaw, Poland | 1:200 | - | - |
| donkey anti-rabbit-Alexa Fluor 647 | #A31573; Thermo Fisher Scientific, Warsaw, Poland | 1:200 | - | - |
| donkey anti-rabbit-Alexa Fluor 488 | #A21206; Thermo Fisher Scientific, Warsaw, Poland | 1:200 | - | - |
| rabbit anti-Akt1/2/3 (H-136) | sc-8312; Santa Cruz Biotechnology Inc., Heidelberg, Germany | - | 1:200 | - |
| rabbit anti-phospho-Akt (Thr 308) (D25E6) | #13038; Cell Signaling Technology, Beverly, MA, USA | - | 1:1000 | - |
| rabbit anti-phospho-Akt1/2/3 (Ser 473) (C-11) | sc-514032; Santa Cruz Biotechnology Inc., Heidelberg, Germany | - | 1:200 | - |
| mouse anti-GAPDH (0411) | #sc-47724; Santa Cruz Biotechnology Inc., Heidelberg, Germany | - | 1:200 | - |
| rabbit anti-phospho-GSK-3α/β (Ser21/9) (D17D2) | #8566; Cell Signaling Technology, Beverly, MA, USA | - | 1:1000 | - |
| mouse anti-GSK-3α/β Antibody (0011-A) | sc-7291; Santa Cruz Biotechnology Inc., Heidelberg, Germany | - | 1:200 | - |
| rabbit anti-ILK | #bs-0317R; Bioss Inc., Woburn, MA, USA | 1:100 | 1:300 | - |
| rabbit anti-ILK | #250718; Abbiotec Inc., Escondido, CA, USA | 1:100 | - | 10 μg/ml |
| mouse anti-Islet 1/2 (39.4D5) | #39.4D5; DSHB, Iowa City, IA, USA | 1:25 | - | - |
| mouse anti-NF-m (4H6) | #4H6; DSHB, Iowa City, IA, USA | 1:5 | - | - |
| normal rabbit IgG | #2027; Santa Cruz Biotechnology, Heidelberg, Germany | - | - | 10 μg/ml |
| normal rabbit IgG | #2729; Cell Signaling Technology, Beverly, MA, USA | - | - | 10 μg/ml |
| HRP-conjugated anti-rabbit | #7074; Cell Signaling Technology, Beverly, MA, USA | - | 1:4000 | - |
| HRP-conjugated anti-mouse | #7076; Cell Signaling Technology, Beverly, MA, USA | - | 1:4000 | - |
| goat anti-Sox10 (N-20) | #sc-17342; Santa Cruz Biotechnology Inc., Heidelberg, Germany | 1:50 | - | - |
| mouse anti-vinculin (V284) | MCA465GA; Bio-rad, Hercules, CA, USA). | - | 1:1000 | - |

## Slide 7
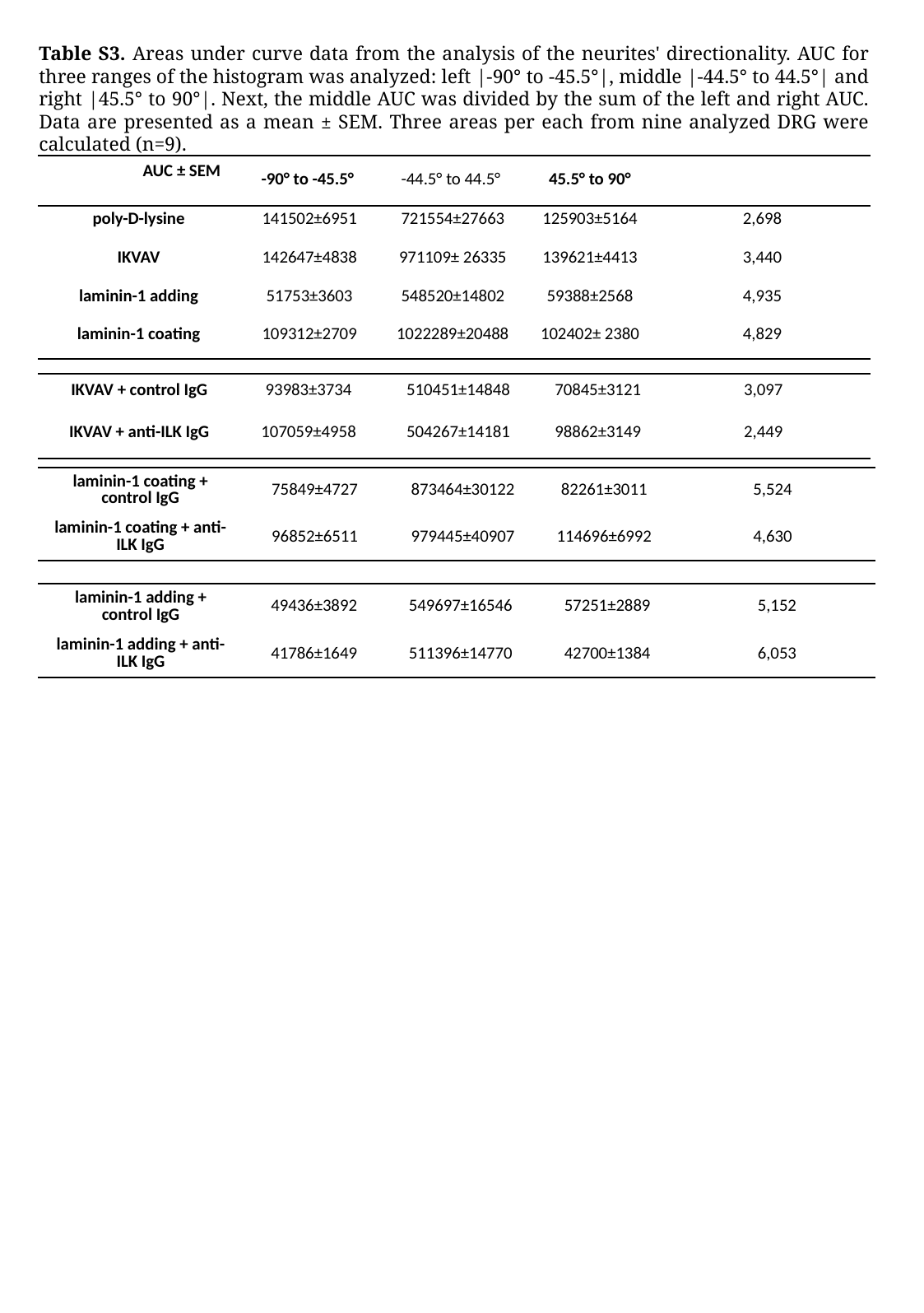

Table S3. Areas under curve data from the analysis of the neurites' directionality. AUC for three ranges of the histogram was analyzed: left |-90° to -45.5°|, middle |-44.5° to 44.5°| and right |45.5° to 90°|. Next, the middle AUC was divided by the sum of the left and right AUC. Data are presented as a mean ± SEM. Three areas per each from nine analyzed DRG were calculated (n=9).
| IKVAV + control IgG | 93983±3734 | 510451±14848 | 70845±3121 | 3,097 |
| --- | --- | --- | --- | --- |
| IKVAV + anti-ILK IgG | 107059±4958 | 504267±14181 | 98862±3149 | 2,449 |
| laminin-1 coating + control IgG | 75849±4727 | 873464±30122 | 82261±3011 | 5,524 |
| --- | --- | --- | --- | --- |
| laminin-1 coating + anti-ILK IgG | 96852±6511 | 979445±40907 | 114696±6992 | 4,630 |
| laminin-1 adding + control IgG | 49436±3892 | 549697±16546 | 57251±2889 | 5,152 |
| --- | --- | --- | --- | --- |
| laminin-1 adding + anti-ILK IgG | 41786±1649 | 511396±14770 | 42700±1384 | 6,053 |
